# HIV incidence and predictors of inconsistent condom use among adult men enrolled into an HIV vaccine preparedness study, Rustenburg, South Africa

**DOI:** 10.1371/journal.pone.0214786

**Published:** 2019-04-03

**Authors:** Pholo Maenetje, Christina Lindan, Heeran Makkan, Candice M. Chetty-Makkan, Mary H. Latka, Salome Charalambous, Mandla Mlotshwa, Matshidiso Malefo, William Brumskine, Nancy K. Hills, Matthew A. Price, Vinodh Edward

**Affiliations:** 1 The Aurum Institute, Johannesburg, South Africa; 2 Advancing Care and Treatment for TB/HIV, A Collaborating Centre of the South African Medical Research Council, Johannesburg, South Africa; 3 School of Pathology, Faculty of Health Sciences, University of the Witwatersrand, Johannesburg, South Africa; 4 Department of Epidemiology and Biostatistics, University of California at San Francisco, San Francisco, United States of America; 5 School of Public Health, Faculty of Health Sciences, University of the Witwatersrand, Johannesburg, South Africa; 6 International AIDS Vaccine Initiative, New York, United States of America; Leibniz Institute for Prevention Research and Epidemiology BIPS, GERMANY

## Abstract

**Introduction:**

Understanding HIV incidence and risk behaviour among populations being considered for HIV vaccine studies is necessary for the appropriate design of trials.

**Methods:**

Between May 2012 and June 2015, we recruited men aged 18–49 years from urban and peri-urban areas of Rustenburg, a mining town in the North West Province, South Africa. Men who reported HIV-risk behaviour were followed for nine to 12 months to determine HIV incidence and factors associated with condom use.

**Results:**

A total of 400 HIV uninfected men were enrolled; 366 (91.5%) had at least one follow-up visit and were included in the analysis; 47.6% were under 25 years of age. HIV incidence was 1.9 per 100 person-years (95% CI: 0.79–4.56). Among heterosexual men (N = 339), 80.8% reported having vaginal intercourse with multiple partners in the past three months, among whom 74.1% reported inconsistent condom use. Sixty-eight percent reported vaginal intercourse with new female partners, of whom 40.6% reported inconsistent condom use. Over half (55.6%) of men who had sex with men (N = 27) reported anal intercourse with multiple male partners in the past three months, of whom 68.2% reported using condoms inconsistently. Men who had more than two female partners in the last three months (n = 121) were more likely to use condoms inconsistently (aOR 4.31, 95% CI: 1.34–13.8); in contrast, those with more than one new female sex partner (aOR 0.13, 94% CI 0.04–0.44), and whose sexual debut was after 19 years of age (aOR 0.39, 95% CI: 0.15–1.01) were less likely to use condoms inconsistently.

**Conclusion:**

HIV incidence was low and similar to other studies of heterosexual men in South Africa. To identify men at high risk for HIV for enrolment in prevention trials, future researchers may need to focus on those who report early sexual debut and who report having multiple sexual partners. Men in newer relationships appear to use condoms more frequently.

## Introduction

South Africa has the highest burden of HIV/AIDS in the world. In 2016, 7.1 million people were estimated to be living with HIV and 270,000 people became newly infected that year [1]. Prevention strategies that have been implemented include promotion of condom use, prevention of maternal-to-child-transmission of HIV, treatment of infected persons with highly active antiretroviral therapy (HAART), voluntary medical male circumcision and pre-exposure prophylaxis (PrEP) [2, 3]. Prospective cohort-based surveys offer precise measurement of HIV incidence and risk factors [4]; however, most estimates of new HIV infections in South Africa have primarily been measured through changes in prevalence over time or laboratory determination of recent infections [5]. In addition, the majority of cohort studies measuring HIV incidence in South Africa have been conducted among women [6–9], with only a few among men who have sex with men (MSM) [10, 11] and heterosexual males [12, 13]. Additional prospective cohort-based information on HIV incidence and risk behaviour among heterosexual men is necessary for the design of HIV vaccine efficacy trials and effective prevention strategies.

Rustenburg is a platinum-mining town in the North West province of South Africa and has a population of approximately 626,522 [14]. HIV prevalence rates are high in this area. In 2012, 20.3% of adult men and women in the province were estimated to be HIV infected [5], and in 2013, 31.5% of antenatal women in and around Rustenburg were estimated to be HIV sero-positive [15]. In two longitudinal cohorts using different recruitment strategies and eligibility criteria, HIV incidence among women in the area was found to be 3.0 per 100 person-years of observation (p100pyo) (95% CI: 0.4–10.8) in 2008–2009 [7], and 8.9 p100pyo (95% CI: 5.7–14.0) in 2010–2011 [10]. Based on a longitudinal cohort study, HIV incidence among MSM, also from the Rustenburg area, was found to be 9.5 p100pyo (95% CI: 2.4–38.0) [10]. However, information on heterosexual men in the Rustenburg area is limited. Therefore, we recruited a cohort of primarily heterosexual men from the same area, and followed them over time to determine HIV incidence and to characterize risk behaviour.

## Methods

### Study subjects

Between May 2012 and June 2015, we recruited men 18–49 years of age from urban and peri-urban areas of Rustenburg for enrolment into a longitudinal observational cohort study. Research staff approached men in the central business district, townships, primary health care clinics, shopping centres, taxi stands, taverns, and car wash stations, as well as during general community meetings and HIV awareness campaigns. Some men heard about the study by word of mouth. Staff briefly explained that the study was recruiting men for a 9-to-12 month follow-up study that would include repeat HIV testing. Men who were interested were asked to come to the research site for evaluation and enrolment. Starting in March 2013, we also began recruiting men by snow-ball sampling in which enrolled participants were each given five coupons to give to their friends or acquaintances, encouraging them to come to the study site for more information and screening. We did not collect information on how enrolled participants were recruited. Participants received mobile phone airtime worth R 30 (2.50 USD) for each referral who was screened.

### Initial HIV testing prior to screening and enrolment

At the study site, staff described the study, and individuals who were interested were asked if they were aware of their HIV status; those who said they were HIV seropositive were excluded. The remaining men underwent risk reduction counselling and were tested for HIV using the Alere Determine^TM^ HIV1-2 rapid test (Alere, USA) on finger prick blood samples; those with a positive result were retested using Uni-gold^TM^ (Trinity Biotech, Ireland). Discordant results were resolved by using an HIV-1 p24Ag ELISA test (HIV Combi PT, Roche Switzerland) on a venous blood sample; the test was performed at Lancet Laboratories, Rustenburg. Men who were HIV seropositive on both rapid tests or by ELISA were referred for care and were not eligible for screening and enrolment. The remaining men were invited to participate in the study and provided signed informed consent in the language of their choice (English, Setswana or Xhosa).

### Screening and enrolment

Screening and enrolment visits were conducted on the same or different days; if the enrolment visit took place more than 28-days after the screening visit, then HIV testing and screening for behavioural risk factors were repeated to ensure continued eligibility.

#### Screening

Participants completed an interviewer-administered questionnaire on socio-demographics and HIV-risk behaviour) during the previous three months (S1–S6 Files) that asked about the following: total number of male and female partners, total number of new male and female partners, condom use with all partners and with new partners (never, sometimes, frequently, always), and self-reported history of symptoms of sexually transmitted infection (STI) (penile discharge, painful urination, dyspareunia or penile sores).We classified inconsistent condom use as never, frequently or sometimes using condoms with partners in the last three months, and consistent condom use as always using a condom with all partners [16, 17]. Clinicians conducted a genital exam; STIs were diagnosed syndromically and treated according to national guidelines. Uncircumcised men were referred to local health care clinics for voluntary medical male circumcision (VMMC). We did not collect information on how many uncircumcised men subsequently underwent VMMC. As part of the study screening procedures, venous blood samples were collected for repeat HIV rapid testing using the same algorithm described above, and those who were identified as HIV infected were referred for care and removed from the study.

#### Enrolment

Eligibility for enrolment included being confirmed as HIV uninfected and reporting high risk behaviour. High risk behaviour was defined as being diagnosed with an STI on exam, or reporting at least one of the following in the last three months: 1) being treated for, diagnosed with, or reporting symptoms of an STI, 2) having vaginal or anal intercourse with more than one sexual partner, 3) having vaginal or anal intercourse with a new sexual partner, or 4) having sex with a partner known to be HIV infected. MSM were defined as men who described their sexual orientation as homosexual or bisexual, and/or who reported having insertive or receptive anal intercourse with another man in the last three months. Men who reported male-male sex and who also reported having sex with women were classified as MSM.

### Follow-up

Detailed contact information was collected at enrolment; participants were asked to return three months later, and again between nine to 12 months after baseline. Men were contacted by phone at six months to update their address and other contact details. At each follow-up visit, men underwent HIV testing of venous blood samples, risk reduction counselling, and a clinical exam for the presence of STIs with treatment as needed. Risk assessment questionnaires were not administered during follow-up visits. Attempts were made to contact men who missed follow-up appointments by phone or through a home visit.

The study was reviewed and approved by the Biomedical Research Ethics Committee of the University of KwaZulu-Natal, and the Research Committee of the Northwest Provincial Department of Health. Men were reimbursed R50 (4.20 USD) at each visit for their time and transport costs.

### Analyses

Data were analysed using STATA V14.0 (Stata Corporation, College Station, Texas, USA). Socio-demographic and behavioural characteristics of heterosexual men (N = 339) were compared to MSM (N = 27) using descriptive statistics. Pearson’s Chi square (χ^2^) and fisher’s exact tests were used to compare proportions (%) for categorical variables and Mann-Whitney *U*-test or Student’s *t*-test was used to compare means and or medians for continuous variables. HIV seroconversion was estimated to have occurred halfway between the date of the last HIV negative test, and the date when HIV antibodies were detected. HIV incidence was calculated as the number of seroconversions divided by the total number of person-years of observation, and presented as p100pyo with 95% confidence intervals (CI). Evaluation of factors associated with HIV incidence was not conducted due to the small number of men who seroconverted.

Logistic regression analysis was used to determine the relationship of socio-demographic and risk-behaviour variables with inconsistent condom use with all types of partners in the last three months (compared to consistent use). We performed univariate logistic regression to evaluate the association of all baseline variables with inconsistent condom use; factors associated with inconsistent condom use at p< = 0.20 were included in a backward stepwise multivariable logistic regression model. Variables with a p-value of <0.05 were retained in the final model; age was included as a potential confounder. All statistical tests were two-sided and p-values <0.05 were considered statistically significant.

## Results

**Fig 1:**
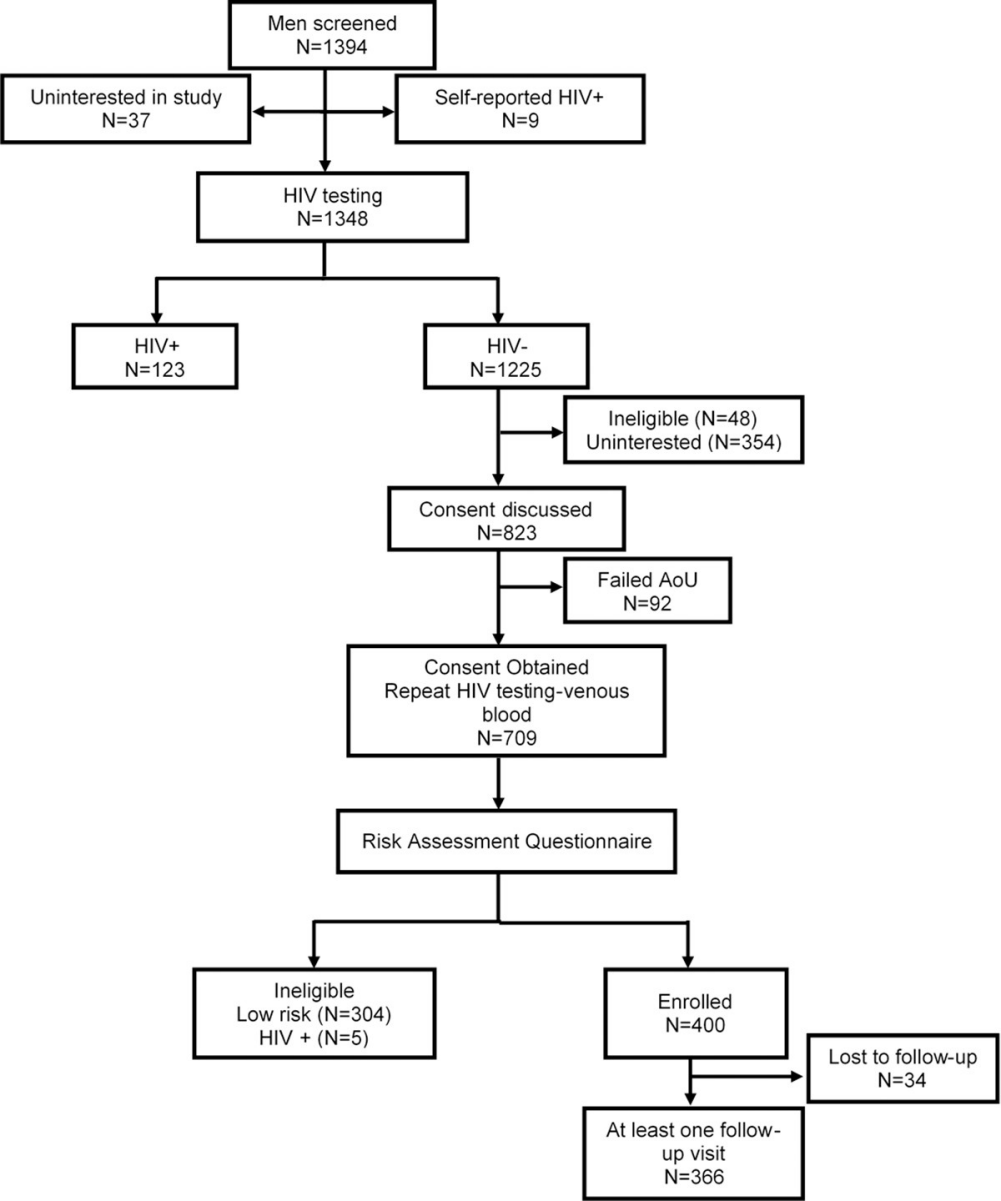
Screening and enrolment of adult men into a longitudinal cohort study, Rustenburg, South Africa, 2012–2015. (AoU)-Assessment of Understanding of the informed consent.

A total of 1394 men presented to the study site for screening, of whom nine (0.6%) reported that they were HIV infected, and 37 (2.6%) decided they were not interested in the study (Fig 1). The remaining 1348 were offered HIV testing and counselling, of whom 123 (9.1%) were identified as being HIV seropositive on testing; 48 were excluded because they preferred a consent language not available or they were not within the target age group; an additional 354 decided not to participate due to work commitments or because they said they were not interested. Staff explained the informed consent to the remaining 823 men, of whom 92 failed an assessment of understanding, and 22 decided not to enrol. The remaining 709 men were screened for eligibility based on risk behaviour, and underwent repeat HIV testing. Of these, 304/709 (42.8%) were ineligible because they did not meet the criteria for being high risk, and five (0.7%) were found to be HIV-infected. The HIV prevalence among the 1394 men who initially presented to the study was 9.8%, including those who reported that they were HIV infected as well as those who were identified as HIV seropositive on testing.

A total of 400 men were enrolled into the cohort and completed baseline data collection of whom 34 (8.5%) did not return for any follow-up visits and were excluded from analysis; thus, 366 men were included in the final cohort. We did not find any significant differences in socio-demographic characteristics and HIV-risk behaviour between men who were retained in the study and those who did not return for follow-up (S1 Table).

### Baseline characteristics

Among the 366 men in the cohort, 27 were classified as being MSM (Table 1). The mean age of all men was 26 (SD 5.5) years; nearly half (47.6%) were less than 25 years of age; 48.9% were unemployed and 53.8% had some post-secondary education. A total of 58 (15.8%) reported being less than 15 years old at the time of their sexual debut; the majority (65.6%) reported becoming sexually active between 15 and 18 years of age. About a third (34.4%) were circumcised. Although 25.1% of men reported having a symptom of an STI in the past three months, only 6.1% were classified as having an STI based on syndromic diagnosis at enrolment. Among men who reported only having heterosexual sex (N = 339), 80.8% had two or more partners in the previous three months, of whom 203/274 (74.1%) reported inconsistent condom use. Nearly three-quarters (68.1%) of heterosexual men reported vaginal intercourse with a new female sexual partner in the past three months, of whom 93/229 (40.6%) reported using condoms inconsistently. Only a few heterosexual men (4.1%) reported sexual intercourse with partners known to be HIV infected; five of these 14 men reported using condoms inconsistently with those partners.

**Table 1:** Baseline socio-demographic and behavioural characteristics of adult HIV negative men enrolled in a longitudinal cohort study between 2012–2015, Rustenburg, South Africa.

			Total (N = 366)		Heterosexual (N = 339)		MSM (N = 27)	
Characteristics			n	(%)	n	(%)	n	(%)
**Age, yrs.**								
** **	Mean (SD)		26.2±5.5		26.5±5.5		21.8±2.5	
**Education**								
** **	Primary school		4	1.1	4	1.2	0	0.0
** **	Some Secondary school		165	45.1	149	44.2	16	59.3
	Some-Post Secondary school		197	53.8	186	55.2	11	40.7
**Employment**								
	Unemployed		179	48.9	167	49.2	12	44.4
	Student		34	9.3	26	7.7	8	29.6
	Employed <40 hrs./wk.		40	10.9	37	10.9	3	11.1
	Employed ≥40 hrs./wk.		113	30.9	109	32.2	4	14.8
**Marital status**								
	Single		347	94.8	321	94.7	26	96.3
	Married		16	4.4	16	4.7	0	0.0
	Divorced/Separated		2	0.5	2	0.6	0	0.0
**Age at sexual debut, yrs.**								
** **	<15		58	15.8	51	15.1	7	25.9
	15–18		240	65.6	224	66.1	16	59.2
	≥19		68	18.5	64	18.8	4	14.8
**Circumcision**								
	Uncircumcised		240	65.5	223	65.7	17	62.9
** **	Medical circumcision		86	23.5	79	23.3	7	25.9
** **	Cultural circumcision		40	10.9	37	10.9	3	11.1
**Self-report of any STI symptoms, last 3 mo.**			92	25.1	89	25.3	3	11.1
**STI by syndromic diagnosis**			22	6.1	22	6.5	0	0.00
**No. female sex partners, last 3 mo.**			** **	** **				
	0		24	6.6	3	0.9	21	77.8
	1		65	17.8	62	18.3	3	11.1
	2		153	41.8	153	45.1	0	0.0
	≥3		124	33.9	121	35.7	3	11.1
*Condom use with female sex partners*, *last 3 mo*. *(n = 342)*								
* *		* *			*n = 336*		*n = 5*	
	Inconsistent		256	74.8	251	74.7	5	83.3
	Consistent		86	25.1	85	25.3	1	16.7
**No. new female sex partners, last 3 mo.**^**a**^ **(n = 352)**								
					*n = 339*		*n = 13*	
	0		118	33.5	108	31.9	10	76.9
	1		151	42.9	150	44.2	1	7.6
	2		46	13.1	46	13.6	0	0.0
	≥3		37	10.5	35	10.3	2	15.4
*Condom use with new female sex partners*, *last 3 mo*.^*a*^ *(n = 232)*								
* *	* *		* *	* *	*n = 229*		*n = 3*	* *
	Inconsistent		95	40.9	93	40.6	2	66.7
	Consistent		137	59.1	136	59.4	1	33.3
**No. male sex partners, last 3 mo.**								
	0		344	18.5	339	100.0	5	18.5
	1		7	1.9	0	0.0	7	25.9
	2		7	1.9	0	0.0	7	25.9
	≥3		8	2.2	0	0.0	8	29.6
*Condom use with male sex partners*, *last 3 mo*. *(n = 22)*								
** **	Inconsistent		15	68.2	-	-	15	68.2
** **	Consistent		7	31.8	-	-	7	31.8
**Had known HIV infected sex partners, last 3 mo.**^**1**^			15	4.2	14	4.1	1	4.0
*Condom use with HIV infected partners*, *last 3 mo*.^*a*^ *(n = 12)*								
* *	* *		*n = 12*		*n = 11*		*n = 1*	
	Inconsistent		6	50.0	5	45.5	1	100.0
	Consistent		6	50.0	6	54.5	0	0.0

^a^ Differences in total numbers among categories are due to missing values.

More than three-quarters (81.5%) of MSM reported having either receptive or insertive anal intercourse with at least one male sexual partner during the past three months, of whom 15/22 (68.2%) reported inconsistent condom use. Over half (55.6%) of MSM reported anal intercourse with two or more male partners in the past three months, of whom 11/15 (73.3%) reported using condoms inconsistently. Less than a quarter of MSM (22.2%) reported having vaginal intercourse with a female in the past three months. Among the 22 MSM who reported recent male-male sexual behaviour, six (27.2%) reported having insertive anal intercourse, of whom three (50.0%) reported inconsistent condom use; 18 (81.8%) reported having receptive anal intercourse, of whom 12 (66.6%) reported inconsistent condom use with these partners (S2 Table).

### HIV incidence and retention

Among the 366 men retained in the study 54 (14.7%) returned for only one follow up visit, and 312 (85.3%) completed both follow-up visits. The median follow-up time was 8.2 months (range: 2.3–23.8), with 263.4 total pyo. Five men became HIV-infected, resulting in an HIV incidence of 1.9 p100pyo (95% CI: 0.8–4.6). Three of the five HIV seroconversions were identified at the final nine or 12 month visit, and the median follow-up time among the five HIV infected men was 9.3 months. All five participants who seroconverted were heterosexual, became sexually active before they were 18 years of age, and had two or more sexual partners in the last three months; only two of the five reported using condoms consistently. None reported having intercourse with a known HIV-infected person. If we exclude the MSM, the total follow-up time among heterosexual men in this study was 243.2 years with an HIV incidence of 2.1 p100pyo (95% CI: 0.84–4.9).

### Inconsistent condom use and socio-demographic factors

Bivariate and multivariable analysis of risk factors associated with inconsistent condom use are shown in Table 2. There was no association between age, being employed and circumcision status and inconsistent use. Men who reported having more than two female sexual partners in the past three months were more likely to inconsistently use condoms (aOR: 4.31; 95% CI: 1.34–13.8). In contrast, men with one or more new female sex partners (aOR: 0.13; 95% CI: 0.04–0.44) were more likely to use condoms. Although not statistically significant, men who had their sexual debut at age ≥ 19 years (aOR: 0.39; 95% CI: 0.15–1.01, p = 0.05) were also more likely to use condoms.

**Table 2:** Association of factors with using condoms inconsistently with all partners in last 3 months, among heterosexual men.

Characteristic		Total (N = 336)	Condom use				Univariate analysis			Multivariable analysis		
			Consistent (n = 85)		Inconsistent (n = 251)							
		N	n	(%)	n	(%)	Unadjusted OR	95% CI	*p*-value	Adjusted OR	95% CI	*p*-value
**Age, yrs.**												
	18–24	151	40	26.5	111	73.5	Ref					
	25–30	120	31	25.8	89	74.2	1.03	0.59–1.78	0.90	1.31	0.71–2.35	0.37
	>30	65	14	21.5	51	78.5	1.31	0.66–2.63	0.44	1.72	0.81–3.63	0.16
**Age at sexual debut, yrs.**												
	<15	51	9	17.6	42	82.4	Ref					
	15–18	222	56	25.3	166	74.7	0.64	0.29–1.39	0.26	0.57	0.26–1.28	0.18
	≥19	63	20	31.7	43	68.3	0.46	0.19–1.13	0.09	0.39	0.15–1.01	**0.05**
**Employment Status**												
	Unemployed	166	44	26.5	122	73.5	Ref					
	Student	25	6	24.0	19	76.0	1.14	0.43–3.04	0.79	-	-	-
	Employed Part-Time	37	10	27.1	27	72.9	0.97	0.43–2.17	0.94	-	-	-
	Employed Full time	108	25	23.2	83	76.8	1.19	0.68–2.10	0.53	-	-	-
**Circumcised**												
	No	258	63	24.4	195	75.6	Ref					
	Yes	78	22	28.2	56	71.7	0.82	0.46–1.45	0.51	-	-	-
**Self-report of any STI symptoms, last 3 mo.**												
	No	248	68	27.4	180	72.3	Ref					
	Yes	88	17	19.3	71	80.7	1.57	0.86–2.86	0.14	-	-	-
**No. of female sex partners, last 3 mo.**												
	1	62	14	22.6	48	77.4	Ref					
	2	153	46	30.1	107	69.9	0.68	0.34–1.35	0.27	0.89	0.42–1.92	0.76
	>2	121	25	20.6	96	79.4	1.12	0.53–2.35	0.76	4.31	1.34–13.8	**0.01**
**No. of new female sex partners, last 3 mo.**												
	0	105	20	19.1	85	80.9	Ref					
	1	150	40	26.7	110	73.3	0.65	0.35–1.18	0.16	0.56	0.28–1.11	0.09
	>1	81	25	30.8	56	69.2	0.53	0.27–1.04	0.06	0.13	0.04–0.44	**0.001**

Odds Ratio (OR) refers to the odds of inconsistently using a condom compared to consistent condom use.

Confidence Interval (CI).

## Discussion

Only a few cohort studies have reported the incidence of HIV infection among heterosexual men in South Africa [12, 13] or in other southern African countries [18, 19]. Here, we report the incidence of HIV infection among men from in and around Rustenburg, a mining town in the North West Province of South Africa. We found the overall incidence to be 1.9 p100pyo, which is similar to national estimates of recent infection among adult males (1.21 p100py; 95% CI: 0.97–1.45) based on use of the limiting-antigen avidity (LAg) assay on HIV-infected samples obtained during a 2012 national population-based survey [5]. Mathematical modelling of data from the 2012 survey resulted in a similar estimate of HIV incidence among men (1.6 p100py [95%CI: 0.6–2.7]) [5].

Our data support the results of other studies in southern Africa that found that heterosexual men have a lower HIV incidence compared to women [5, 12, 13, 19, 20]. Several factors may have biased our incidence estimate, however. Because almost half our cohort were under the age of 25 years, our estimates may not be representative of the overall male population in South Africa; national estimates of HIV prevalence are higher among men aged 25–49 years [5]. At each study visit, participants received risk reduction counselling which may have reduced their subsequent risk behaviour; however, we did not collect data on behaviour at follow up visits to see if they changed over time. On the other hand, some studies suggest that impact of counselling on STI and/or HIV acquisition may be limited [21]. The short median follow-up time in our study (8.2 months) may not have been long enough to identify many new HIV infections, although HIV incidence is often highest early in cohort participation [10]. As not all men were retained, it is possible that men at higher risk of acquiring HIV dropped out. However, we found no significant differences in the demographics and HIV-risk behaviour between men who were retained and those who were not. Because of stigma associated with HIV, it is possible that high risk men may have under-reported their HIV-risk behaviour at screening and therefore were considered ineligible to participate in our study. Treatment of STIs and male circumcision may also have contributed to reduced risk of HIV acquisition. Finally, since the research clinic was open only during working hours, men who were employed and could have been at higher risk may have declined enrolment; nearly 50% of men in our sample were unemployed.

To better understand characteristics of men at high risk for HIV infection, we examined the relationship between socio-demographic and behavioural factors, and inconsistent condom use. Similar to previous studies, we observed that men who had multiple female sexual partners were least likely to use condoms [22], although those who had new female sexual partners were more likely to use condoms. Previous studies have shown that men in new/casual relationships are more likely to use condoms, but as relationships become more stable, condom use declines [23–25]. We also observed that those who had earlier sexual debut were less likely to use condoms consistently. Previous studies have shown that males who had their first sexual debut before the age 15 age were less likely to engage in unprotected sex compared to those who had early sexual debut [26]. Based on these observations, future researchers may need to focus on men who report early sexual debut, who have multiple sexual partners and/or men in who are in longer term relationships to identify men at high risk for HIV infection.

Our study has several limitations in addition to those described above. Identification of men based on self-report of risk behaviour may have been subject to recall bias or mis-reporting due to stigma. Studies among adult men and women in Uganda, and among women in South Africa found low HIV incidence rates even though individuals reported engaging in high HIV risk behaviour [7, 27, 28]. Additional studies are needed to determine how best to recruit high risk heterosexual men; possibilities include identifying men from hot-spots or based on their social and sexual network characteristics [29, 30]. We did not collect data on whether uncircumcised men underwent circumcision after being referred by study staff. Because our recruitment strategy targeted men who thought they were HIV-uninfected, we may have inadvertently pre-selected men who were at lower risk. Even though we attempted to recruit men at high risk, 9.8% of those who presented at the clinic were identified as being HIV infected, which is lower than the prevalence of 14.5% among the general adult male population (15–49 years of age) in South Africa in 2012 [5]

This study adds important information to the limited body of knowledge regarding the incidence of HIV infection among heterosexual men in South Africa. Our findings suggests that vaccine efficacy trials that aim to include heterosexual South African men will require more specific recruitment strategies and better characterization of behavioural risk profiles to recruit men at risk of HIV. Results from our study suggest enrolling those with multiple partners and those whose sexual debut occurred when young. In addition, the eligibility criteria for enrolment may need to include whether condoms were used with their sexual partners.

## Supporting information

S1 Table Comparison of socio-demographic and behavioural characteristics between men retained in the study and those lost to follow-up. (DOCX)

S2 Table Sexual behaviour of study participants who reported having male-male sex in the last three months. (DOCX)

S1 File Questionnaire 1_English. (PDF)

S2 File Questionnaire 2_English. (PDF)

S3 File Questionnaire 1_Setswana. (PDF)

S4 File Questionnaire 2_Setswana. (PDF)

S5 File Questionnaire 1_Xhosa. (PDF)

S6 File Questionnaire 2_Xhosa. (PDF)
