# Supplementary material for: HIV incidence and predictors of inconsistent condom use among adult men enrolled into an HIV vaccine preparedness study, Rustenburg, South Africa
Source: PLoS One. 2019 Apr 3;14(4):e0214786. doi: 10.1371/journal.pone.0214786 (PMC6447216; doi:10.1371/journal.pone.0214786)
Supplement: S1 Table — (DOCX) [file pone.0214786.s001.docx]

**Supplementary Table 1. Comparison of socio-demographic and behavioural characteristics between men retained in the study and those lost to follow-up.**

|  | | **Total** | | **Retained Participants** | | **Lost to Follow-up** | | **p-value** |
| --- | --- | --- | --- | --- | --- | --- | --- | --- |
|  | | **(N=400)** | | **(N=366)** | | **(N=34)** | |  |
| **Characteristics** | | **n** | **(%)** | **n** | **(%)** | **n** | **(%)** |  |
| **Age, yrs.** | | | | | | | |  |
|  | Mean (SD) | 26.2±5.5 | | 26.5±5.5 | | 27.8±2.5 | |  |
| **Education** | | | | | | | |  |
|  | Primary school | 5 | 1.3 | 4 | 1.1 | 1 | 2.9 | 0.65 |
|  | Some Secondary school | 180 | 45.0 | 165 | 45.1 | 15 | 44.1 |  |
|  | Some-Post Secondary school | 215 | 53.7 | 197 | 53.8 | 18 | 52.9 |  |
| **Employment** | | | | | | | |  |
|  | Unemployed | 199 | 49.7 | 179 | 48.9 | 20 | 58.8 | 0.63 |
|  | Student | 37 | 9.3 | 34 | 9.3 | 3 | 8.8 |  |
|  | Employed <40 hrs./wk. | 44 | 11.0 | 40 | 10.9 | 4 | 11.7 |  |
|  | Employed ≥40 hrs./wk. | 120 | 30.0 | 113 | 30.8 | 7 | 20.9 |  |
| **Marital status** | | | | | | | |  |
|  | Single | 377 | 94.4 | 347 | 95.1 | 30 | 88.2 | 0.15 |
|  | Married | 2 | 0.5 | 2 | 0.5 | 0 | 0.0 |  |
|  | Divorced/Separated | 20 | 5.1 | 16 | 4.4 | 4 | 11.7 |  |
| **Age at sexual debut, yrs.** | | | | | | | |  |
|  | <15 | 63 | 15.7 | 58 | 15.8 | 5 | 14.7 | 0.57 |
|  | 15-18 | 265 | 66.2 | 240 | 65.5 | 25 | 73.5 |  |
|  | ≥19 | 72 | 18.0 | 68 | 18.5 | 4 | 11.7 |  |
| **Circumcision** | |  |  |  |  |  |  |  |
|  | Uncircumcised | 263 | 65.7 | 240 | 65.5 | 23 | 67.6 | 0.11 |
|  | Medical circumcision | 90 | 22.5 | 86 | 23.5 | 4 | 11.7 |  |
|  | Cultural circumcision | 47 | 11.7 | 40 | 10.9 | 7 | 20.6 |  |
| **Self-report of any STI symptoms, last 3 mo.** | | 99 | 24.8 | 92 | 25.1 | 7 | 21.2 | 0.61 |
| **STI by syndromic diagnosis** | | 23 | 5.7 | 22 | 6.1 | 1 | 2.9 | 0.46 |
| **No. female sex partners, last 3 mo.** | | | | | | | | |
|  | 0 | 25 | 6.3 | 24 | 6.5 | 1 | 2.9 | 0.61 |
|  | 1 | 69 | 17.3 | 65 | 17.7 | 4 | 11.7 |  |
|  | 2 | 168 | 42.0 | 153 | 41.8 | 15 | 44.2 |  |
|  | ≥3 | 138 | 34.5 | 124 | 33.8 | 14 | 41.2 |  |
| *Condom use with female sex partners, last 3 mo. (n=375)* | | | | | | | |  |
|  |  |  | | *n=342* |  | *n=33* |  |  |
|  | Inconsistent | 282 | 75.2 | 256 | 74.8 | 26 | 78.8 | 0.62 |
|  | Consistent | 93 | 24.8 | 86 | 25.2 | 7 | 21.2 |  |
| **No. new female sex partners, last 3 mo. (n=386)** | | | | | | | |  |
|  |  |  |  | *n=352* |  | *n=34* |  |  |
|  | 0 | 128 | 33.2 | 118 | 33.5 | 10 | 29.4 | 0.42 |
|  | 1 | 168 | 43.5 | 151 | 42.9 | 17 | 50.0 |  |
|  | 2 | 52 | 13.5 | 46 | 13.1 | 6 | 17.6 |  |
|  | ≥3 | 38 | 9.8 | 37 | 10.5 | 1 | 2.9 |  |
| *Condom use with new female sex partners. last 3 mo^a^ (n=255)* | | | | | | | |  |
|  |  |  |  | *n=232* | | *n=23* |  |  |
|  | Inconsistent | 104 | 40.8 | 95 | 40.9 | 9 | 39.2 | 0.86 |
|  | Consistent | 151 | 59.2 | 137 | 59.1 | 14 | 60.8 |  |
| **No. male sex partners, last 3 mo.** | | | | | | | |  |
|  | 0 | 378 | 94.5 | 344 | 93.9 | 34 | 100.0 | 0.53 |
|  | 1 | 7 | 1.7 | 7 | 1.9 | 0 | 0.0 |  |
|  | 2 | 7 | 1.7 | 7 | 1.9 | 0 | 0.0 |  |
|  | ≥3 | 8 | 2.0 | 8 | 2.2 | 0 | 0.0 |  |
| *Condom use with male sex partners. last 3 mo. (n=22)* | | | | | | | |  |
|  | Inconsistent | 15 | 68.2 | 15 | 68.2 | 0 | 0.0 | N/A |
|  | Consistent | 7 | 31.8 | 7 | 31.8 | 0 | 0.0 |  |
| **Had known HIV infected sex partner, last 3 mo.^a^** | | 15 | 3.7 | 15 | 4.2 | 0 | 0.0 | 0.2 |
| *Condom use with HIV infected partners. last 3 mo.^a^ (n=12)* | | | | | | | |  |
|  | Inconsistent | 6 | 50.0 | 6 | 50.0 | 0 | 0.0 | N/A |
|  | Consistent | 6 | 50.0 | 6 | 50.0 | 0 | 0.0 |  |

^a^ Differences in total numbers among categories are due to missing values.
