# Supplementary material for: HIV incidence and predictors of inconsistent condom use among adult men enrolled into an HIV vaccine preparedness study, Rustenburg, South Africa
Source: PLoS One. 2019 Apr 3;14(4):e0214786. doi: 10.1371/journal.pone.0214786 (PMC6447216; doi:10.1371/journal.pone.0214786)
Supplement: S2 Table — (DOCX) [file pone.0214786.s002.docx]

**Supplementary Table 2. Sexual behaviour of study participants who reported having male-male sex in the last three months.**

|  |  |  | **MSM (n=22)** | |
| --- | --- | --- | --- | --- |
| **Characteristics** | | | **n** | **(%)** |
| **Receptive anal sex, last 3 mo.** | |  |  |  |
|  | No |  | 4 | 18.1 |
|  | Yes |  | 18 | 81.8 |
| *Condom use during receptive anal sex, last 3 mo.(n=18)* | | | | |
|  | Inconsistent | | 12 | 66.6 |
|  | Consistent | | 6 | 33.3 |
| **Insertive anal sex, last 3 mo.** | |  |  |  |
|  | No |  | 18 | 72.7 |
|  | Yes |  | 6 | 23.3 |
| *Condom use during insertive anal sex, last 3 mo. (n=6)* | | | | |
|  | Inconsistent | | 3 | 50.0 |
|  | Consistent | | 3 | 50.0 |
