# Supplementary material for: HIV incidence and predictors of inconsistent condom use among adult men enrolled into an HIV vaccine preparedness study, Rustenburg, South Africa
Source: PLoS One. 2019 Apr 3;14(4):e0214786. doi: 10.1371/journal.pone.0214786 (PMC6447216; doi:10.1371/journal.pone.0214786)
Supplement: S1 File — (PDF) [file pone.0214786.s003.pdf]

Date: ___ ___ /___ ___ ___ / ___ ___ ___ ___
DD / MON / Y Y Y Y **Put 5 digit PIN here →** ___________________

Start time: ________:______

Aurum Screening Questionnaire

- **Thank you for agreeing to take part in our research.**

- **We are interested in knowing about people's health. Some of these questions are sensitive, but it is very important that we know the truth about what is going on in this community. There are no right or wrong answers. Please be honest when answering these questions.**

- **<u>You do not have to answer any questions if you don't want to</u> – what is most important is that you feel comfortable answering honestly.**

- **Your answers to these questions will tell us about the community which will help us for future research projects.**

- **All of your answers will be kept confidential. Confidential means we will not tell your answers to anyone outside the research group.**

Date: ___ ___ /___ ___ ___ / ___ ___ ___ ___
DD / MON / Y Y Y Y **Put 5 digit PIN here → __________________**

Research Assistant]			**CODE**
D1	**How did you first hear about the study? Please pick <u>one</u> of the options from this card.**	1= In a clinic<br>2= Approached in my neighborhood<br>3= Through mobile VCT<br>4= Through a community meeting or out in community<br>5= Heard about it from a friends / family<br>6= From my partner<br>7= From school<br>8= Walk in<br>9= Tavern/shebeen/ place where alcohol is consumed<br>10= Other (specify below)	
D2	If code 10 above,,write where they heard about Aurum from here →		
D3	**How old are you?**	Code in years→	
D4	**What is your date of birth?**	Write in date ___ ___ / ___ ___ ___ / ___ ___ ___ ___<br>DAY / M O N / Y Y Y Y	
D5	**In which area of Rustenburg do you live?** Write area here→		
D6	**What is your gender? That is, are you male, female, or transgender or transsexual?**	0= Male<br>1= Female<br>2= Transgender/ transsexual	
D7	**How would you describe your employment situation? Please pick from this card.**	0= Unemployed looking for work<br>1= Unemployed not looking for work<br>2= Unable to work - receive social grant<br>3= Student/pupil/learner<br>4= Self-employed – part time less than 40 hours per week<br>5= Self-employed – full time 40 hours or more per week<br>6= Employed, less than 40 hours per week<br>7= Employed, full time (40 hours or more)<br>8= Other	
D7o	If code 8 above, write employment situation→		
D8	**How would you describe your family's financial situation?**	1= Not enough money for basic things like food, clothes<br>2= Have money for food and clothes but short on many other things<br>3= We have the basics but not enough money for expensive things<br>4= Have money to save or buy expensive things<br>5= Other	
D8o	If code 5 above, write family situation →		
D9	**Compared with <u>other famili</u>es would you say that your family is financially better off, about the same, or poorer than others?**	0= Poorer than others<br>1= About the same as others<br>2= Better off than others	
D10	**Compared with <u>your peers</u> would you say that you are financially better off, about the same, or poorer than your peers?**	0= Poorer than others<br>1= About the same as others<br>2= Better off than others	

Date: ___ ___ /___ ___ ___ / ___ ___ ___ ___
DD / MON / Y Y Y Y **Put 5 digit PIN here → __________________**

			CODE
D11<br>DEM2 8	**What has been your <u>primary source of income</u> in the last 12 months? (code up to 2 choices: first choice here, second choice in next question)**	1= Family<br>2= Formal employment<br>3= Casual labour<br>4= Spouse, boyfriend, girlfriend<br>5= Self-employment<br>6= None<br>7= Other	
D12<br>DEM2 8o	If code 7 above Write primary source of income here→		
D13<br>DEM2 8	**Any other main source of income?**	1= Family<br>2= Formal employment<br>3= Casual labour<br>4= Spouse, boyfriend, girlfriend<br>5= Self-employment<br>6= None<br>7= Other	
D14<br>DEM28o	If code 7 above Write primary source of income here→		
D15<br>DEM2 9	**Are you currently caring (financially) for any dependents?**	0= No<br>1=Yes	
D16	**How would you describe your relationship status?**<br><br>**[Pick from card]**	1= Married (civil magistrate /traditional /religious)<br>2= Not married but living together<br>3= Single but have a steady partner<br>4= Single but have steady and casual partner(s)<br>5= Single but have casual partner(s)<br>6= Single with no partners<br>7= Other	
D16o	**If code 7 above**	Write relationship status here→	
D17	**Does your partner live with you?**	0= No<br>1=Yes	
D18	**<u>[If has only one partner of any kind]</u>**<br>**How long have you been with this partner?**	Code in months for all time together. Take out time not together. Code 0 if one time partner →	
D19	**<u>[If any partners]</u>**<br>**In the last month, how often have you seen your partner(s)?**	1= Less than once a month<br>2= About once a month<br>3= About two to three times a month<br>4= Once a week<br>5= More than once a week	
D20	**Please tell me which of these statements best describes your sexual orientation?**	0= Heterosexual<br>1= Homosexual<br>2= Bisexual<br>3= Heterosexual but sometimes have sex with the same sex<br>4= Homosexual but sometimes have sex with the opposite sex<br>5= No orientation, I sleep with whomever I like	

77= refused 88= don't know (if not already listed) 97= not applicable 99= missing data

Date: ___ ___ /___ ___ ___ / ___ ___ ___ ___
DD / MON / Y Y Y Y **Put 5 digit PIN here → __________________**

Now some questions about you

				CODE
DEM11	**What is your race?**	1= Black<br>2= White<br>3= Asian<br>4= Other (specify below)		
DEM11o	Write in other here→			
DEM12	**What is your tribal or ethnic group?**	Write in name here →		
DEM 13	**What is your country of birth?**	Write in name here →		
**How many total years of school have you completed in each category?**				
DEM14a	**a. Primary**		Code number of years →	
DEM14b	**b. Secondary**		Code number of years →	
DEM 14c	**c. Post-secondary**		Code number of years →	
DEM14d	**d. Completed years of other school/apprenticeship**		Code number of years →	
DEM14e	**If has other school/apprenticeship, specify type here→**			
DEM15	**What is your religion?**		1= Catholic<br>2= Protestant<br>3= Other Christian<br>4= Muslim<br>5= None<br>6= Other	
DEM15o	If above is other specify other religion here→			
DEM16	**How many years have you lived in this area**	Code number of years; code= 00 if less than 1 year →		
DEM17	**What is your current marital status?**	1= Single<br>2= Divorced/separated<br>3= Married, monogamous<br>4= Married, polygamous<br>4= Widowed		

77= refused 88= don't know (if not already listed) 97= not applicable 99= missing data

Date: ___ ___ /___ ___ ___ / ___ ___ ___ ___
DD / MON / Y Y Y Y   Put 5 digit PIN here → __________________

Now some questions about things you may or may not own. These questions refer to things that <u>you personally own</u> (not about your household).

	**Do you personally own a…**	**USE THESE CODES**<br>**0= No**<br>**1= Yes**
PP1	**...working wrist watch?**	
PP2	**…jewelry?** (ring, necklace, chains made of gold, silver platinum etc.)	
PP3	**…working cell phone?** (any type)	
PP4	**…working cell phone that has a camera?**	
PP5	**….working cell phone that connects to internet?** (Facebook, Twitter, MXIT)	
PP6	**…working iPod / personal music device / MP3 player?**	
PP7	**...working computer?** (laptop/desktop; any kind of working computer=yes)	
PP8	**…working iPad**	
PP9	**…working private car**	
PP10	**…working taxi or similar vehicle using to commute passengers**	
PP11	**…working motor bike /scooter?**	
PP12	**… a house – either alone or jointly with someone else?**	
PP13	**... any land – either alone or jointly with someone else?**	
PP14	**… a cheque or saving account at a <u>bank</u>?**	
PP15	**... a <u>bank</u> credit card?**	
PP16	**…a loan from a cash loan store?**	
PP17	**… any kind of store credit card?**	

			**CODE**
PP18	**How often do you save money?**	**0**=Not saving<br>1= Save once a year (includes 13th cheque)<br>2= Every <u>six months</u> or so<br>3= Every <u>few months</u><br>4= Save <u>every</u> month	

77= refused 88= don't know (if not already listed) 97= not applicable 99= missing data

Date: ___ ___ /___ ___ ___ / ___ ___ ___ ___
DD / MON / Y Y Y Y **Put 5 digit PIN here → __________________**

Now let's talk about things you may or may not have in your household. When I ask about household I am talking about the place where you mainly stay right now– not about your home village or where you come from. For these questions, I am talking about things that are in working condition.

	**Does your household have a working…**	**USE THESE CODES**<br>**0= No**<br>**1= Yes**
HH1	**… TV?** (any kind)	
HH2	**… plasma or flat screen TV?**	
HH3	**…DSTV, MNET or TopTV, or other satellite television connection?**	
HH4	**…an entertainment system / sound system?**	
HH5	**…working fridge?**	
HH6	**…working landline telephone?**	
HH7	**…working electricity?**	
HH8	**…working water tap in house?**	
HH9	**…working water tap in yard?**	

Now I am going to read you a list about supporting others. Please tell me how often you have done any of these things in the last 3 months.

	**In the last 3 months, how often did you…**	**READ CODES OUT LOUD TO PARTICIPANT**<br>0=Never<br>1= A few times<br>2= Monthly or more
FS1	**…buy your family food for the house?**	
FS2	**…give food, money or things to support your family household?**	
FS3	**…give food, money or things to support other family members (outside your immediate household)?**	
FS4	**…give food, money or things to support friends?**	
FS5	**…give food, money or things to help support a lover (who is not part of your household as per above)?**	
FS6	**…give food, money or things to help support a someone you want as a lover?**	

77= refused 88= don't know (if not already listed) 97= not applicable 99= missing data

Date: ___ ___ /___ ___ ___ / ___ ___ ___ ___
DD / MON / Y Y Y Y **Put 5 digit PIN here → __________________**

ASK OFALL—REGARDLESS OF ANY ANSWERS GIVEN ABOVE ABOUT EMPLOYEMENT

Now I'm going to ask you some questions about your working situation. You do not have to answer any question that you don't want to. There are no right or wrong answers to these questions. Please be honest when you answer. Like the census, we are asking because we want to better understand this community.

E1	**What is your job? That is, what kind of work do you do?**	Write in job here and be as descriptive as possible. If no job write not working	
E2	**How many hours, including overtime, did you work during the <u>last 7 days?</u>**	Code number of hours worked (if did not work, code =0)→	**CODE**
E3	**How many hours <u>per week</u>, including overtime, do you <u>usually</u> work?**	Code number of hours worked (if did not work, code =0)→	
E4	**Do you want to work longer hours than you currently are working?**	0= No<br>1= Yes	
E5	**Do you usually work throughout the year, or do you work seasonally, or only once in a while?**	0= Work once in a while<br>1= Works seasonally<br>2= Works throughout the year	
E6	**How are you paid for work done, or are you not paid at all?**	0= Not paid<br>1= Paid in kind only (get things/services not money)<br>2= Paid in cash and kind<br>3= Paid in cash only	
E7	**How much job security do you feel you have?**	0= No job security<br>1= Some job security<br>2= Pretty good job security<br>3= Very secure job	
E8	**Please tell me the letter that matches your total monthly pay for your main job (include overtime, allowances, bonuses)?**<br><br>**[hand out card, read options before asking question]**	In Rands,<br>A. 0 – 2,000<br>B. 2,001 – 5,000<br>C. 5,001 – 10,000<br>D. 10,001 – 15,000<br>E. 15,001 – 20,000<br>F. More than 20,000	**Code letter here**

Date: ___ ___ /___ ___ ___ / ___ ___ ___ ___
DD / MON / Y Y Y Y **Put 5 digit PIN here → __________________**

**Now let's talk about your social life. Please tell me how often you have done these thing in the last 3 months.** <br>**[hand out card, read options before asking questions]**		
	**In the last 3 month how often did you…**	**READ CODES OUTLOUD TO PARTICIPANT**<br><br>0=Never<br>1=Rarely (have done, but not regularly)<br>2= Sometimes (do this from time to time)<br>3= Often (I do this weekly)<br>4= Regularly (do this more than 1 time per week)
SL1	**…buy a prepared meal at a tuckshop, take away or restaurant for yourself?**	
SL2	**…buy a prepared meal at a tuckshop, take away or restaurant for family members?**	
SL3	**…buy a prepared meal at a tuckshop, take away or restaurant for your friends?**	
SL4	**…buy a prepared meal at a tuckshop, take away or restaurant for your lovers?**	
SL5	**…buy a prepared meal at a tuckshop, take away or restaurant for someone you want as a lover?**	
SL6	**...give gifts/things other than food/drink to a lover?**	
SL7	**...give giftst/things other than food/drink to someone you want as a lover?**	
SL8	**…spend time at a tavern/ shebeen?**	
SL9	**…spend time hanging out at shopping centers/ hair salons/ or tuckshops?**	
SL10	**… spend time card playing or gambling?**	
SL11	**…spend time at informal car washes or picnic spots?**	
SL12	**…buy yourself a drink at a tavern/ shebeen/ place to drink alcohol?**	
SL13	**…buy your friends a drink at a tavern/ shebeen/ place to drink alcohol?**	
SL14	**…buy a someone you want as a lover a drink at a tavern / shebeen/ place to drink alcohol?**	

77= refused 88= don't know (if not already listed) 97= not applicable 99= missing data

Date: ___ ___ /___ ___ ___ / ___ ___ ___ ___
DD / MON / Y Y Y Y **Put 5 digit PIN here → __________________**

			**CODE**
RIS11	**<u>During the last month</u>, on average, how often have you had a drink containing alcohol?**	0= None<br>1= 1-3 times per month<br>2= Weekly<br>3= Daily	
RIS12	**<u>During the last month</u>, how often were you drunk/drinking alcohol before sex?**	0= Never<br>1= Sometimes (less than half)<br>2= Frequently (more than half)<br>3= Always	
****<br>**Some people have tried a range of different types of drugs. <u>In the last month</u>, which of the following, if any, have you used?**			
RIS13a	**Khat/ Miraa**	0= No<br>1= Yes<br>88= don't know	
RIS13b	**Injecting drugs with a needle**	0= No<br>1= Yes<br>88= don't know	
RIS13c	**Marijuana**	0= No<br>1= Yes<br>88= don't know	
RIS13d	**Other**	0= No<br>1= Yes<br>88= don't know	
RIS13do	Specify other drug use here →		

Date: ___ ___ /___ ___ ___ / ___ ___ ___ ___
DD / MON / Y Y Y Y **Put 5 digit PIN here → __________________**

[For women only ask:] Now some questions about your family and reproductive history. (For males code=97)

Now some questions about family life…			CODE
PG1	**Have you ever been pregnant?**	0 = No<br>1 = Yes	
PG2	**If yes, how old were you when you fell pregnant the first time?**	Code age as 97 if never pregnant.	
PG3	**Are you pregnant right now?**	0 = No<br>1 = Yes<br>2 = Not sure	
PG4<br>DEM210a	**How many total past pregnancies have you had?**	WRITE number →<br>USE IAVI rules for NA.	
PG5	**How many of these pregnancies were planned?**	WRITE number here→<br>97 if never pregnant.	
PG6<br>DEM210b	**How many live births have you had?**	WRITE number here→<br>USE IAVI rules for NA.	
PG7	**When was the last time you gave birth to a living child?**	WRITE Mo & Yr here→	**_____ /_____**<br>**MON / YYYY**
PG8<br>DEM210c	**How many of your children died between the ages of 0 and 5 years of age?**	WRITE number here→<br>USE IAVI rules for NA	
PG9	**How many biological children do you have? That means your own children.**	WRITE number here→	
PG10	**Are you satisfied with the number of children that you currently have?**	1 = Very unsatisfied<br>2 = Slightly unsatisfied<br>3= No feelings / neutral<br>4 = Satisfied<br>5 = Very satisfied	
PG11	**How many children in total would you like to have?**	WRITE number here→	
PG12	**How many different biological fathers are there for your children?**	WRITE number here→<br>97 if never pregnant.	
PG13	**In the last 3 months, have you or your partner wanted to fall pregnant?**<br><br>**[Read options if needed]**	1 = I wanted to, but my partner didn't<br>2 = I did not want to, but my partner did<br>3 = Both wanted to<br>4 = Both did not want to	
PG14	**Do you or your partner want to fall pregnant in the next year?**<br><br>**[Read out options]**	1 = I want to, but my partner doesn't<br>2 = I do not want to, but my partner does<br>3 = We both want to<br>4 = We both do not want to	
PG15	**What is the best age for a female to fall pregnant for the first time?**	WRITE age here→	
PG16	**What is the best age for a man to father a child?**	WRITE age here→	
PG17	**How old were you when you first had sex (vaginal or anal)?**	WRITE age here→	

Date: ___ ___ /___ ___ ___ / ___ ___ ___ ___
DD / MON / Y Y Y Y **Put 5 digit PIN here →** ________________

[For men only ask:] Now some questions about your family and reproductive history. (For females code=97)

			CODE
PG1	**Have you ever gotten a woman/ girl pregnant?**	0 = No<br>1 = Yes<br>2= Not sure	
PG2	**If yes, how old were you when you got someone pregnant the first time?**	Code 99 if never created pregnancy.	
PG3	**Are any of your partners pregnant right now?**	0 = No<br>1 = Yes<br>2 = Not sure	
PG4	**How many pregnancies have you been responsible for (no matter the outcome)?**	WRITE number here→<br>97 if never created a pregnancy.	
PG5	**How many of these pregnancies were planned?**	WRITE number here→<br>97 if never created a pregnancy.	
PG6	**How many live births have you been the biological father to?**	WRITE number here→<br>97 if never created a pregnancy.	
PG7	**When was the last time you fathered a living child?**	WRITE Mo&Yr here→	_____ /_____<br>**MON / YYYY**
PG8	**How many of your children died between the ages of 0 and 5 years of age?**	WRITE number here→<br>97 if never fathered a child	
PG9	**How many biological children do you have? That means your own children.**	WRITE number here→	
PG10	**Are you satisfied with the number of children that you currently have?**	1 = Very unsatisfied<br>2 = Slightly unsatisfied<br>3= No feelings / neutral<br>4 = Satisfied<br>5 = Very satisfied	
PG11	**What is your ideal number of children?**	WRITE number here→	
PG12	**How many different biological mothers are there for your children?**	WRITE number here→<br>97 if never fathered a child	
PG13	**In the last 3 months, have you or your partner wanted to fall pregnant? [Read options if needed]**	1 = I wanted to, but my partner didn't<br>2 = I did not want to, but my partner did<br>3 = Both wanted to<br>4 = Both did not want to	
PG14	**Do you or your partner want to fall pregnant in the next year? [Read out options]**	1 = I want to, but my partner doesn't<br>2 = I do not want to, but my partner does<br>3 = We both want to<br>4 = We both do not want to.	
PG15	**What is the best age for a female to fall pregnant for the first time?**	WRITE age here→	
PG16	**What is the best age for a male to father a child?**	WRITE age here→	
PG17	**How old were you when you first had sex (vaginal or anal)?**	WRITE age here→	

Date: ___ ___ /___ ___ ___ / ___ ___ ___ ___
DD / MON / Y Y Y Y **Put 5 digit PIN here → __________________**

Now I'm going to ask you about your beliefs. People have many different beliefs. First, I would like to hear your beliefs about condoms. I am going to read you a statement, please tell me how much you agree or disagree with this statement using a number from this card. Remember there are no right or wrong answers. Please tell me your opinion.

[Hand out card; explain how to use it]

		Strongly disagree	Disagree	Agree	Strongly Agree
CU1	**I am able to convince my partner to use condoms even if s/he or she does not want to.**	**1**	**2**	**3**	**4**
CU2	**I would remember to use a condom even after I have been drinking.**	**1**	**2**	**3**	**4**
CU3	**I could stop to put a condom on myself or my partner even if we are both very sexually aroused.**	**1**	**2**	**3**	**4**
CU4	**I can refuse sex when there is no condom available.**	**1**	**2**	**3**	**4**
CU5	**If I were to suggest using condoms with a partner that I hadn't been using condoms with, I would feel afraid that s/he would reject me.**	**1**	**2**	**3**	**4**
CU6	**If I suggest using condoms with a new partner then s/he would think I have a sexually transmitted disease.**	**1**	**2**	**3**	**4**
CU7	**If I suggest using condoms with a new partner then s/he would think I thought they had a sexually transmitted disease.**	**1**	**2**	**3**	**4**

Now some questions about your beliefs related to pregnancy. Please tell me how much you agree or disagree with the following statements. [Repeat instructions and reminders]

	[Provide response card]	Strongly disagree	Disagree	Agree	Strongly Agree
PB1	**A girl must fall pregnant to show that she is fertile.**	1	2	3	4
PB2	**A man will only want to marry a girl if she has been made pregnant by him before.**	1	2	3	4
PB3	**A girl is not a woman unless she has had a child.**	1	2	3	4
PB4	**A boy is not a man until he has had a child with a woman.**	1	2	3	4
PB5	**It is a family shame if a girl falls pregnant before being married.**	1	2	3	4
PB6	**A man will only want to marry a girl if she has borne his child/children.**	1	2	3	4
PB7	**Parents get a smaller lobola if their daughter was pregnant and had a child.**	1	2	3	4
PB8	**Females fall pregnant so that they can obtain a government child grant.**	1	2	3	4
PB9	**A woman believes that she must fall pregnant to show that she is a real woman.**	1	2	3	4
PB10	**Friends influence each other to fall pregnant.**	1	2	3	4
PB11	**Pregnancy is a sign of femininity.**	1	2	3	4
PB12	**Men believe that a woman must fall pregnant to show that she is a real woman.**	1	2	3	4

77= refused 88= don't know (if not already listed) 97= not applicable 99= missing data

Date: ___ ___ /___ ___ ___ / ___ ___ ___ ___
DD / MON / Y Y Y Y **Put 5 digit PIN here → ________________**

Now some questions about your beliefs about relationships between men and women.

	**[Provide response card]**	**Strongly Disagree**	**Disagree**	**Agree**	**Strongly Agree**
R1	Men have many lovers because it is in their nature to do so	1	2	3	4
R2	Men have lovers to get energy to satisfy their primary partners	1	2	3	4
R3	Women these days say that they need to have more than one sex partner	1	2	3	4
R4	Men feel ashamed of their wives and want young lovers to take around to their friends	1	2	3	4
R5	If men do not have lovers their friends laugh at them	1	2	3	4
R6	Women who are financially independent do not want to commit themselves to one relationship	1	2	3	4
R7	The families of young people who work do not want them to get married because they are afraid to lose their income	1	2	3	4
R8	Men often force women in subtle ways to have sex with them even if they do not want to	1	2	3	4
**A husband is justified in hitting or beating his wife if she…**					
PWR1	…goes out without telling him.	1	2	3	4
PWR2	…neglects the children.	1	2	3	4
PWR3	…argues with him.	1	2	3	4
PWR4	… refuses to have sex with him.	1	2	3	4
PWR5	…burns the food.	1	2	3	4

Finally, some questions about ways to prevent HIV.

		**CODE** 0= No 1= Yes 2= Not sure
K1	Is there currently a vaccine that stops people from getting infected with HIV?	
K2	Is there currently a vaccine that slows down disease after people are infected with HIV?	
K3	Is there currently a gel that women can put in their vaginas and stop them from getting HIV during sex?	
K4	If man has had his penis circumcised by a doctor is he less likely to get infected with HIV?	

This is the end of the questionnaire. Thank you for taking the time to answer these questions.

TIMEEND	**Time Interview Ended**	HH:MM using 24 hour clock	___ ___ : ___ ___

[Refer as needed. Correct knowledge as needed using text below]:

- I want to make sure that you know that there is currently no vaccine to stop or slow down HIV.
- There is currently no gel product that women can put in their vaginas to stop them from getting HIV during sex.
- Vaccines and gels are being tested in South Africa to find out if they work. But right now we do not have a vaccine or a gel that works.
- Men who have had their penises circumcised by a doctor are at lower risk of getting HIV. I can give you the contact details of a doctor who does circumcision.

Date: ___ ___ /___ ___ ___ / ___ ___ ___ ___
DD / MON / Y Y Y Y **Put 5 digit PIN here →** ________________

INFORMATION TO BE COMPLETED BY INTERVIEWER AFTER INTERVIEW:

				CODE
I1	**Interviewer code for interviewer**			
I2	**In what language was the interview administered?**	0=English<br>1=Tswana<br>2=Afrikaans<br>3=Mix of English & Tswana<br>4=Xhosa<br>5=Zulu<br>6=Other		
I2o		If other specify→		

INFORMATION TO BE COMPLETED BY STUDY COORDINATOR OR DESIGNEE:

		**CODE**
**Is the participant eligible for IAVI Protocol B?**	0= Ineligible<br>1= Eligible<br>97= Not applicable	
