# Supplementary material for: HIV incidence and predictors of inconsistent condom use among adult men enrolled into an HIV vaccine preparedness study, Rustenburg, South Africa
Source: PLoS One. 2019 Apr 3;14(4):e0214786. doi: 10.1371/journal.pone.0214786 (PMC6447216; doi:10.1371/journal.pone.0214786)
Supplement: S3 File — (PDF) [file pone.0214786.s005.pdf]

Date: ___ ___ /___ ___ ___ / ___ ___ ___ ___
DD / MON / Y Y Y Y **Put 5 digit PIN here →** ________________

Start time: ________:______

Pampiri ya Dipotso ya Aurum ya go Bona Gore a Motho o a Tshwanelega

- **Re lebogela gore o bo o dumetse go nna le seabe mo patlisisong ya rona.**

- **Re rata go itse ka pholo ya batho. Dingwe tsa dipotso di ka nna tsa go tlhabisa ditlhong go di araba, lefa go ntse jalo go botlhokwa thata gore re itse boammaaruri kaga se se diragalang mo lefelong leno la baagi. Ga go na dikarabo tse di siameng kgotsa tse di phoso. Tsweetswee araba dipotso ka boikanyegi.**

- **<u>Ga o patelesege go araba dipotso dipe fela fa o sa batle go di araba -</u> selo sa botlhokwatlhokwa ke gore o ikutlwe o phuthologile fa o araba dipotso ka boikanyegi.**

- **Dikarabo tsa gago mo dipotsong tseno di tlile go re bolelela ka baagi ba mono eleng se se tlileng go re thusa mo diporojekeng tsa rona tsa dipatlisiso tsa mo isagweng.**

- **Dikarabo tsa gago tsotlhe di tlile go bolokiwa e le khupamarama. Khupamarama e kaya gore ga re tle go bolelela ope yo o seng mo setlhopheng sa patlisiso eno dikarabo tsa gago.**

Date: ___ ___ /___ ___ ___ / ___ ___ ___ ___
DD / MON / Y Y Y Y **Put 5 digit PIN here → __________________**

Mothusi wa mo Patlisisong			CODE
D1	**O utlwaletse patlisiso eno kae lekgetlo la ntlha? Tsweetswee tlhopha karabo e le nngwe mo karateng eno.**	1= Mo tliliniking<br>2= Mongwe wa baagelani o mpoleletse ka yone<br>3= Go tswa go VCT e e tsamayang<br>4= Kwa kopanong ya baagi kgotsa go tswa mo baaging<br>5= Ke e utlwaletse go tswa mo ditsaleng / lelapeng<br>6= Go tswa mo mothong yo ke ratanang le ene<br>7= Go tswa kwa sekolong<br>8= Ke itletse fela<br>9= Kwa thabeneng/kwa šibining/ kwa lefelong le go nwelwang bojalwa kwa go lone<br>10= Tse dingwe (tlhalosa fa tlase fano)	
D2	If code 10 above,,write where they heard about Aurum from here →		
D3	**O na le dingwaga tse kae?**	Code in years→	
D4	**O tsetswe ka letlha lefe?**	Write in date<br>___ ___ / ___ ___ ___ / ___ ___ ___ ___<br>LETSATSI / KGWEDI / NGWAGA	
D5	**O nna mo tulong efe ya Rustenburg?** Write area here→		
D6	**A o monna kgotsa o mosadi? Ke raya gore, a o monna, mosadi, kgotsa a o fetotse bong jwa gago?**	0= Monna<br>1= Mosadi<br>2= Ke fetotse bong	
D7	**O ka tlhalosa jang maemo a o leng mo go one ka tiro? Tsweetswee tlhopha mo karateng eno.**	0= Ga ke bereke mme ke batla tiro<br>1= Ga ke bereke, ga ke a tshwaragana le go batla tiro<br>2= Ga ke kgone go bereka – ke amogela madi a puso<br>3= Ke moithuti/morutwa/morutwana<br>4= Ke a ipereka – ke bereka tiro ya nakwana ya diura tse di kafa tlase ga di le 40 ka beke<br>5= Ke a ipereka – ke bereka tiro ya letsatsi lotlhe ya diura di le 40 kgotsa go feta ka beke<br>6= Ke a bereka, tiro ya diura tse di kafa tlase ga di le 40 ka beke<br>7= Ke bereka, tiro ya letsatsi lotlhe (diura di le 40 kgotsa go feta ka beke)<br>8= Tse dingwe	
D7o	If code 8 above, write employment situation→		
D8	**O ne o ka tlhalosa jang maemo a lelapa la gago le leng mo go one mo go tsa ditšhelete?**	1= Ga re na madi a a lekaneng go ka reka dilo tsa botlhokwa tsa go tshwana le dijo, diaparo<br>2= Re na le madi a go ka reka dijo le diaparo, mme fela re tlhaela madi a go reka dilo tse dingwe tse dintsi<br>3= Re na le dilo tsa botlhokwa lefa go ntse jalo ga re na madi a go reka dilo tse di turang<br>4= Re na le madi a re ka a bolokang kgotsa go reka dilo tse di turang<br>5= Tse dingwe	
D8o	If code 5 above, write family situation →		
D9	**Fa o tshwantsha lelapa la lona le <u>malapa a mangwe</u> a o ne o ka re lelapa la lona le botoka mo go oneka madi, le batla le tshwana**	0= Le humanegile go gaisiwa ke mangwe	

77= ganne 88= ga ke itse (if not already listed) 97= ga e maleba 99= tshedimosetso e e tlhaelang

	**le one, kgotsa le humanegile go feta malapa a mangwe?**	1= Le batla le tshwana le a mangwe<br>2= Le gaisa a mangwe	
D10	**Fa o itshwantsha le <u>ditsala tsa gago</u> a o ne o ka re o botoka mo go bone mo ditšheleteng, o batla o tshwana le bone, kgotsa o humanegile go feta ditsala tsa gago?**	0= Le humanegile go gaisiwa ke mangwe<br>1= Le batla le tshwana le a mangwe<br>2= Le gaisa a mangwe	

				CODE
D11<br>DEM2 8	**Motswedi o mogolo wa madi a o a amogelang e ne e le eng mo dikgweding tse 12 tse di fetileng? (code up to 2 choices: first choice here, second choice in next question)**		1= Lelapa<br>2= Tiro ya ka gale<br>3= Tiro e e seng ya ka gale<br>4= Mogatsake, lekau la me, kgarebe ya me<br>5= Ke a ipereka<br>6= Ga go na dipe<br>7= Tse dingwe	
D12<br>DEM2 8o	If code 7 above Write primary source of income here→			
D13<br>DEM2 8	**A go na le selo sepe fela se sengwe se segolo se lo bonang madi a lotseno mo go sone?**		1= Lelapa<br>2= Tiro ya ka gale<br>3= Tiro e e seng ya ka gale<br>4= Mogatsake, lekau la me, kgarebe ya me<br>5= Ke a ipereka<br>6= Ga go na dipe<br>7= Tse dingwe	
D14<br>DEM28o	If code 7 above Write primary source of income here→			
D15<br>DEM2 9	**A go na le bangwe ba o ba tlhokomelang (ka madi) ga jaana?**		0= Nnyaa<br>1=Ee	
D16	**O ka tlhalosa jang maemo a gago a thatano?**<br><br>**[Pick from card]**	1= Ke nyetse/nyetswe (kwa go mmagiseterata/ka setso/ka sedumedi)<br>2= Ga ke a nyala/nyalwa mme fela ke nna le mongwe<br>3= Ga ke a nyala/nyalwa lefa go ntse jalo ke na le molekane yo o tlhoafetseng<br>4= Ga ke a nyala/nyalwa lefa go ntse jalo ke na le (ba)motho yo ke ratanang le ene yo o tlhomameng le wa go tsamaisa nako<br>5= Ga ke a nyala/nyalwa lefa go ntse jalo ke na le (ba)molekane wa go tsamaisa nako<br>6= Ga ke a nyala/nyalwa mme ga ke na ope yo ke ratanang le ene<br>7= Tse dingwe		
D16o	**If code 7 above**	Write relationship status here→		
D17	**A motho yo o ratanang le ene o nna le wena?**	0= Nnyaa<br>1=Ee		
D18	**<u>[If has only one partner of any kind]</u>**<br>**O na le nako e e kana kang o ratana le motho yono?**	Code in months for all time together. Take out time not together. Code 0 if one time partner →		
D19	**[Fa a na le balekane]**	1= Kafa tlase ga gangwe ka kgwedi		

	**O bone (ba)molekane wa gago ga kae mo kgweding e e fetileng?**	2= Mo e ka nnang gangwe ka kgwedi<br>3= Mo e ka nnang gabedi kgotsa gararo ka kgwedi<br>4= Gangwe ka beke<br>5= Go feta gangwe ka beke	
D20	**Tsweetswee mpolelela gore ke polelo efe mo go tseno e e tlhalosang mokgwa wa gago wa go robalana?**	0= Ke robalana le batho ba bong jo bo farologaneng le jwa me<br>1= Ke robalana le batho ba bong jo bo tshwanang le jwa me<br>2= Ke robalana le batho ba bong jo bo farologaneng le jwa me mmogo le ba bong jo bo tshwanang le jwa me<br>3= Ke robalana le batho ba bong jo bo farologaneng le jwa me lefa go ntse jalo ka dinako tse dingwe ke robalana le batho ba bong jo bo tshwanang le jwa me<br>4= Ke robalana le batho ba bong jo bo tshwanang le jwa me lefa go ntse jalo ka dinako tse dingwe ke robalana le batho ba bong jo bo farologaneng le jwa me<br>5= Ga ke na mokgwa wa go robalana, ke robalana le mongwe le mongwe fela yo ke mo ratang	

Date: ___ ___ /___ ___ ___ / ___ ___ ___ ___
DD / MON / Y Y Y Y **Put 5 digit PIN here →** ________________
Jaanong go latela dipotso di sekae kaga gago

				CODE
DEM11	**O mokae?**	1= Montsho<br>2= Mosweu<br>3= Moasia<br>4= Tse dingwe (tlhalosa fa tlase fano)		
DEM11o	Write in other here→			
DEM12	**O wela mo setlhopheng sefe sa morafe?**	Write in name here →		
DEM 13	**O tsaletswe kwa nageng efe?**	Write in name here →		
**O tsene sekolo dingwaga tse kae mo setlhopheng sengwe le sengwe?**				
DEM14a	**a. Sekolo sa poraemari**	Code number of years →		
DEM14b	**b. Sekolo sa sekontari**	Code number of years →		
DEM 14c	**c. Sekolo sa morago ga sa sekontari**	Code number of years →		
DEM14d	**d. Dingwaga tse o di feditseng o tsena sekolo sa mofuta o mongwe/o thapisediwa tiro**	Code number of years →		
DEM14e	**If has other school/apprenticeship, specify type here→**			
DEM15	**O tsena kereke efe?**	1= Katoliki<br>2= Porotesetanta<br>3 = E nngwe ya Bokeresete<br>4= Moseleme<br>5= Ga ke tsene epe<br>6= Tse dingwe		
DEM15o	If above is other specify other religion here→			
DEM16	**O na le dingwaga tse kae o nna mo tulong eno?**	Code number of years; code= 00 if less than 1 year →		
DEM17	**A o nyetse/nyetswe ga jaana?**	1= Ga ke a nyala/nyalwa<br>2= Ke tlhadile/tlhadilwe/re kgaogane<br>3= Ke nyetse/nyetswe, mosadi/monna a le mongwe<br>4= Ke nyetse/nyetswe, lefufa<br>4= Ke motlholagadi/moswagadi		

77= ganne 88= ga ke itse (if not already listed) 97= ga e maleba 99= tshedimosetso e e tlhaelang

Date: ___ ___ /___ ___ ___ / ___ ___ ___ ___
DD / MON / Y Y Y Y **Put 5 digit PIN here → ________________**

**Jaanong go latela dipotso di sekae tse di kaga dilo tse o ka tswang o na le tsone kgotsa o sena tsone. Dipotso tseno di malebang le dilo tse eleng tsa gago ka sebele (ga di kaga lelapa la gago).**		
**A wena ka namana o na le...**		**USE THESE CODES**<br>**0= Nnyaa**<br>**1= Ee**
PP1	**...watšhe e e rwalwang mo letsogong e e berekang?**	
PP2	**...dibenyane?** (palamonwana, nekeleisi, ditšheine tse di dirilweng ka gauta, selefera, polatinamo, jalo le jalo)	
PP3	**...selefounu e e berekang?** (mofuta ope fela)	
PP4	**...selulafounu e e berekang e e nang le khamera?**	
PP5	**...selulafounu e e berekang e e kgona go gokagana le inthanete?** (Facebook, Twitter, MXIT)	
PP6	**...iPod e e berekang / selo se se tshamekang mmino sa gago ka namana / setshameka-MP3?**	
PP7	**...khomputara e e berekang?** (laptop/desktop; khomputara ya mofuta ope fela e e berekang=ee)	
PP8	**...iPad e e berekang**	
PP9	**...koloing ya gago ka namana e e berekang**	
PP10	**...tekesi e e berekang kgotsa koloi ya go tshwana le yone e e dirisediwang go rwala bapalami**	
PP11	**...sethuthuthu /sekutara se se berekang?**	
PP12	**...ntlo - e ka tswa e le ya gago o le esi kgotsa e o e tlhakanetseng le mongwe o sele?**	
PP13	**...lefatshe lepe fela - e ka tswa e le la gago o le esi kgotsa le o le tlhakanetseng le mongwe o sele?**	
PP14	**... akhaonto ya tšheke kgotsa ya go boloka madi kwa bankeng?**	
PP15	**... karata ya keretiti ya banka?**	
PP16	**…madi a kadimo go tswa kwa lebenkeleng le le adimisang madi?**	
PP17	**… mofuta ope fela wa karata ya keretiti ya lebenkele?**	

			**CODE**
PP18	**O boloka madi gantsi go le kana kang?**	**0**=Ga ke boloke madi<br>1= Ke a boloka gangwe ka ngwaga (seno se akaretsa tšheke ya bo-13)<br>2= Mo dikgweding dingwe le dingwe tse di ka nnang thataro<br>3= Mo dikgweding dingwe le dingwe di sekae<br>4= Ke a boloka kgwedi le kgwedi	

77= ganne 88= ga ke itse (if not already listed) 97= ga e maleba 99= tshedimosetso e e tlhaelang

Date: ___ ___ /___ ___ ___ / ___ ___ ___ ___
DD / MON / Y Y Y Y **Put 5 digit PIN here → __________________**

Jaanong a re bue ka dilo tse o ka tswang o na le tsone kgotsa o sena tsone mo legaeng la lona. Fa ke go botsa ka legae ke bua ka lefelo le o nnang kwa go lone segolo jaanong jaana - eseng ka legae la gago la kwa magaeng kgotsa kwa o tswang teng. Mo dipotsong tseno, ke bua ka dilo tse di berekang.

	**A lelapa la lona le na le...e e berekang...**	**USE THESE CODES 0= Nnyaa 1= Ee**
HH1	**... thelebišene?** (ya mofuta ope fela)	
HH2	**… thelebišene ya plasma kgotsa ya sekerini se se sephara?**	
HH3	**…DSTV, MNET kgotsa TopTV, kgotsa thelebišene e nngwe e e golaganang ka satalaete?**	
HH4	**…setshamekammino tsa go intsha bodutu / setshamekammino se se dirang modumo o montsi?**	
HH5	**…foritšhi e e berekang?**	
HH6	**…mogala o eseng wa selulafounu o o berekang?**	
HH7	**…motlakase o o berekang?**	
HH8	**…tepe ya metsi ya mo ntlong e e berekang?**	
HH9	**…tepe ya metsi ya mo jarateng e e berekang?**	

Jaanong ke tlile go go balela lenaane fano leo le leng kaga go thusa ba bangwe. Ke kopa gore o mpolelele gore o dirile sepe fela sa dilo tseno gantsi go le kana kang mo dikgweding tse 3 tse di fetileng.

	**Mo dikgweding tse 3 tse di fetileng, ke gantsi go le kana kang o…**	**READ CODES OUT LOUD TO PARTICIPANT** 0=Ga ke ise ke dirise ope 1= Makgetlo a sekae 2= Kgwedi le kgwedi kgotsa go feta moo
FS1	**...reketseng lelapa la lona dijo tsa mo ntlong?**	
FS2	**...fileng legae la lelapa la lona dijo, madi kgotsa dilo go le thusa?**	
FS3	**...fileng maloko a mangwe a lelapa dijo, madi kgotsa dilo go ba thusa (a eseng karolo ya legae la gago)?**	
FS4	**...fileng ditsala dijo, madi kgotsa dilo go ba thusa?**	
FS5	**...fileng motho yo o ratanang le ene dijo, madi kgotsa dilo go mo thusa (yo eseng karolo ya legae la lona go ya ka se se fa godimo)?**	

Date: ___ ___ /___ ___ ___ / ___ ___ ___ ___
DD / MON / Y Y Y Y   Put 5 digit PIN here → ________________

FS6	**...fileng mongwe yo o batlang go ratana le ene dijo, madi kgotsa dilo go mo thusa?**	

ASK OFALL—REGARDLESS OF ANY ANSWERS GIVEN ABOVE ABOUT EMPLOYEMENT

Jaanong ke tlile go go botsa dipotso di sekae kaga mmereko wa gago. Ga o patelesege go araba potso epe fela e o sa batleng go e araba. Ga go na dikarabo tse di siameng kgotsa tse di phoso mo dipotsong tseno. Ke kopa gore fa o araba o dire jalo ka boikanyegi. Go tshwana le fa go balwa batho, re botsa dipotso ka gonne re batla go tlhaloganya lefelo leno la baagi botoka.

E1	**O dira tiro efe? Ke raya gore, o dira tiro ya mofuta mang?**	Write in job here and be as descriptive as possible. If no job write not working	
E2	**O berekile diura tse kae, go akaretsa le nako ya diura tsa morago ga mmereko, mo malatsing a le 7 a a fetileng?**	Code number of hours worked (if did not work, code =0)→	**CODE**
E3	**Ke diura tse kae ka beke, go akaretsa le nako e e fetang ya mmereko, tse o di berekang ka gale?**	Code number of hours worked (if did not work, code =0)→	
E4	**A o batla go bereka diura tse di ditelejana go feta tse o di berekang ga jaana?**	0= Nnyaa<br>1= Ee	
E5	**A gantsi o bereka ngwaga yotlhe, kgotsa a o bereka ka ditlha tse di rileng, kgotsa a o bereka ka sewelo?**	0= O bereka sewelo<br>1= O bereka tiro e e tsamaisanang le ditlha<br>2= O bereka ngwaga yotlhe	
E6	**O duelwa ka tsela efe mo tirong e o e dirileng, kgotsa a ga o duelwe gotlhegotlhe?**	0= Ga ke duelwe<br>1= Ke duelwa go nteboga ka sengwe fela (ka go fiwa dilo/ditirelo eseng madi)<br>2= Ke duelwa madi a kheše le ka go lebogiwa ka sengwe<br>3= Ke duelwa madi a kheše fela	
E7	**O ikutlwa o sireletsegile go le kana kang kgatlhanong le go latlhegelwa ke mmereko?**	0= Ga ke ikutlwe ke sireletsegile kgatlhanong le go latlhegelwa ke mmereko<br>1= Ke ikutlwa ke sireletsegile go sekae kgatlhanong le go latlhegelwa ke mmereko<br>2= Ke ikutlwa ke sireletsegile thatathata kgatlhanong le go latlhegelwa ke mmereko<br>3= Ke ikutlwa ke sireletsegile thata kgatlhanong le go latlhegelwa ke mmereko	
E8	**Ke kopa gore o mpolelele gore ke tlhaka efe e e tshwanelang palogotlhe ya madi a o a amogelang kgwedi le kgwedi kwa tirong ya gago ya konokono (go akaretsa le tiro e o e dirang morago ga diura tsa tiro, dituelotlaleletso, dibonase)?**	Ka Diranta,<br>A. 0 – 2,000<br>B. 2,001 – 5,000<br>C. 5,001 – 10,000<br>D. 10,001 – 15,000	**Code letter here**

	**[hand out card,<br>read options before asking question]**	E. 15,001 – 20,000<br><br>F. Ba ba fetang 20,000	

Date: ___ ___ /___ ___ ___ / ___ ___ ___ ___
DD / MON / Y Y Y Y **Put 5 digit PIN here → _________________**

**Jaanong a re bue ka botshelo jwa gago le ditsala. Ke kopa gore o mpolelele gore o dirile dilo tseno gantsi go le kana kang mo dikgweding tse 3 tse di fetileng.**<br><br>**[hand out card, read options before asking questions]**

	**Mo dikgweding tse 3 tse di fetileng, ke gantsi go le kana kang o…**	**READ CODES OUTLOUD TO PARTICIPANT**<br><br>0=Ga ke ise ke dire sepe<br>1=Ka sewelo (ke se dirile, mme eseng ka metlha)<br>2= Ka dinako tse dingwe (ke dira seno nako le nako)<br>3= Gantsi (ke dira seno beke le beke)<br>4= Ka metlha (ke dira seno go feta gangwe ka beke)
SL1	**...rekileng dijo tse di apeetsweng ruri kwa thakašopong, kwa lefelong la dijo tse o tsamayang ka tsone kgotsa kwa resetšhurenteng o di ithekela?**	
SL2	**...rekileng dijo tse di apeetsweng ruri kwa thakašopong, kwa lefelong la dijo tse o tsamayang ka tsone kgotsa kwa resetšhurenteng o di rekela ba lelapa la gago?**	
SL3	**...rekileng dijo tse di apeetsweng ruri kwa thakašopong, kwa lefelong la dijo tse o tsamayang ka tsone kgotsa kwa resetšhurenteng o di rekela ditsala tsa gago?**	
SL4	**...rekileng dijo tse di apeetsweng ruri kwa thakašopong, kwa lefelong la dijo tse o tsamayang ka tsone kgotsa kwa resetšhurenteng o di rekela batho ba o ratanang le bone?**	
SL5	**...rekileng dijo tse di apeetsweng ruri kwa thakašopong, kwa lefelong la dijo tse o tsamayang ka tsone kgotsa kwa resetšhurenteng o di rekela motho yo o batlang go ratana le ene?**	
SL6	**...fileng motho yo o ratanang le ene dimpho/dilo tse dingwe kwantle ga dijo/dino?**	
SL7	**...fileng motho yo o batlang go ratana ene dimpho/dilo tse dingwe kwantle ga dijo/dino?**	
SL8	**...sentseng nako kwa thabeneng/ šibining?**	
SL9	**...sentseng nako o iphokisa mowa kwa ditikatikweng tsa mabenkele/ kwa disaluneng tsa moriri/ kgotsa kwa dithakašopong?**	
SL10	**... sentseng nako o tshameka dikarata kgotsa o kembola?**	

77= ganne 88= ga ke itse (if not already listed) 97= ga e maleba 99= tshedimosetso e e tlhaelang

Date: ___ ___ /___ ___ ___ / ___ ___ ___ ___
DD / MON / Y Y Y Y **Put 5 digit PIN here → ________________**

SL11	**...sentseng nako kwa mafelong a go tlhatswetsa dikoloi a eseng a semmuso kgotsa kwa dipikiniking?**	
SL12	**...itheketseng sengwe sa go nwa kwa thabeneng/ kwa šibining/ kwa lefelong la go nwa bojalwa?**	
SL13	**...reketseng ditsala tsa gago sengwe sa go nwa kwa thabeneng/ kwa šibining/ kwa lefelong la go nwa bojalwa?**	
SL14	**...reketseng motho yo o batlang go ratana le ene sengwe sa go nwa kwa thabeneng/ kwa šibining/ kwa lefelong la go nwa bojalwa?**	

			CODE
RIS11	**Mo kgweding e e fetileng, ka palogare, o nole seno se se nang le tagi gantsi go le kana kang?**	0= Ga a dirisa epe<br>1= Ga 1-3 ka kgwedi<br>2= Beke le beke<br>3= Letsatsi le letsatsi	
RIS12	**Mo kgweding e e fetileng, ke gantsi go le kana kang o neng o tagilwe/o ileng wa nwa bojalwa pele ga o robalana?**	0= Ga ke ise ke dirise ope<br>1= Ka dinako tse dingwe (kafa tlase ga halofo)<br>2= Kgapetsakgapetsa (go feta halofo)<br>3= Ka metlha	
**Batho ba bangwe ba lekile mefuta e e farologaneng ya diokobatsi. Mo kgweding e e fetileng, o dirisitse sefe sa dilo tse di latelang, fa o di dirisitse?**			
RIS13a	**Khat/ Miraa**	0= Nnyaa<br>1= Ee<br>88= ga ke itse	
RIS13b	**Go itlhaba ka diokobatsi o dirisa nnalete**	0= Nnyaa<br>1= Ee<br>88= ga ke itse	
RIS13c	**Matekwane**	0= Nnyaa<br>1= Ee<br>88= ga ke itse	
RIS13d	**Tse dingwe**	0= Nnyaa<br>1= Ee<br>88= ga ke itse	
RIS13do	Specify other drug use here →		

Date: ___ ___ /___ ___ ___ / ___ ___ ___ ___
DD / MON / Y Y Y Y **Put 5 digit PIN here → __________________**

[For women only ask:] Jaanong ke tlile go go botsa dipotso di sekae ka lelapa la lona le hisitori ya tsalo. (For males code=97)

Jaanong go latela dipotso di sekae kaga botshelo jwa lelapa…			CODE
PG1	**A o setse o kile wa ithwala?**	0 = Nnyaa<br>1 = Ee	
PG2	**Fa o rile ee, o ne o na le dingwaga tse kae fa o ithwala ka lekgetlo la ntlha?**	Code age as 97 if never pregnant.	
PG3	**A o ithwele jaaka re bua jaana?**	0 = Nnyaa<br>1 = Ee<br>2 = Ga ke itse sentle	
PG4<br>DEM210a	**O ithwele palogotlhe ya makgetlo a le kae mo nakong e e fetileng?**	WRITE number →<br>USE IAVI rules for NA.	
PG5	**Ke makgetlo a le kae mo go ano a o ithweleng ka one a o neng o rulaganyeditse seo mo go one?**	WRITE number here→<br>97 if never pregnant.	
PG6<br>DEM210b	**O tsetse bana ba ba tshelang ba le kae?**	WRITE number here→<br>USE IAVI rules for NA.	
PG7	**O feleditse leng go belega (tshola) ngwana yo o tlang a tshela?**	WRITE Mo & Yr here→	_____ /_____<br>**MON / YYYY**
PG8<br>DEM210c	**O na le bana ba le kae ba ba tlhokafetseng ba na le dingwaga tse di magareng ga 0 le 5?**	WRITE number here→<br>USE IAVI rules for NA	
PG9	**O na le bana ba le kae ba eleng ba gago ka tsalo? Seo se raya bana ba eleng ba gago ka sebele.**	WRITE number here→	
PG10	**A o kgotsofaletse palo ya bana ba o nang le bone ga jaana?**	1 = Ga ke a kgotsofala gotlhegotlhe<br>2 = Ga ke a kgotsofala go sekae<br>3= Ga ke na maikutlo / ke fa gare<br>4 = Ke kgotsofetse<br>5 = Ke kgotsofetse thata	
PG11	**O ka rata go nna le palogotlhe ya bana ba le kae?**	WRITE number here→	
PG12	**Bana ba gago ba na le borraabone ba le kae ba ba farologaneng ba tlholego?**	WRITE number here→<br>97 if never pregnant.	
PG13	**Mo dikgweding tse 3 tse di fetileng, a wena kgotsa molekane wa gago o ile a/wa batla go ithwala?**<br><br>**[Read options if needed]**	1 = Ke ne ke batla, mme motho yo ke ratanang le ene o ne a sa batle<br>2 = Ke ne ke sa batle, mme motho yo ke ratanang le ene o ne a batla<br>3 = Re ne re batla roobedi<br>4 = Re ne re sa batle roobedi	
PG14	**A wena kgotsa motho yo o ratanang le ene o batla go ithwala mo ngwageng e e tlang?**<br><br>**[Read out options]**	1 = Ke a batla, mme motho yo ke ratanang le ene ga a batle<br>2 = Ga ke batle, mme motho yo ke ratanang le ene o a batla<br>3 = Re a batla roobedi<br>4 = Ga re batle roobedi	
PG15	**Ke dingwaga dife tse di gaisang tsotlhe tse**	WRITE age here→	

77= ganne 88= ga ke itse (if not already listed) 97= ga e maleba 99= tshedimosetso e e tlhaelang

	**mosadi a tshwanetseng go itlhwala lekgetlo la ntlha ka tsone?**		
PG16	**Ke dingwaga dife tse di gaisang tsotlhe tse monna a tshwanetseng go nna le ngwana ka tsone?**	WRITE age here→	
PG17	**O ne o na le dingwaga tse kae fa o robalana lekgetlo la ntlha (o dirisa bosadi kgotsa sebono)?**	WRITE age here→	

<u>[For men only ask:]</u> Jaanong ke tlile go go botsa dipotso di sekae ka lelapa la lona le hisitori ya tsalo. (For females code=97)

			**CODE**
PG1	**A o setse o kile wa ithwadisa mosadi/ mosetsana?**	0 = Nnyaa<br>1 = Ee<br>2= Ga ke itse sentle	
PG2	**Fa o rile ee, o ne o na le dingwaga tse kae fa o ithwadisa mongwe ka lekgetlo la ntlha?**	Code 99 if never created pregnancy.	
PG3	**A go na le bape ba batho ba o ratanang le bone ba ba ithweleng jaaka re bua jaana?**	0 = Nnyaa<br>1 = Ee<br>2 = Ga ke itse sentle	
PG4	**Ke makgetlo a le kae a o ithwadisitseng batho mo go one (go sa kgathalesege gore dipholo tsa seo e nnile eng)?**	WRITE number here→<br>97 if never created a pregnancy.	
PG5	**Ke makgetlo a le kae mo go ano a o ithweleng ka one a o neng o rulaganyeditse seo mo go one?**	WRITE number here→<br>97 if never created a pregnancy.	
PG6	**Ke bana ba le kae ba ba tsetsweng ba tshela ba o leng rraabone wa tlholego?**	WRITE number here→<br>97 if never created a pregnancy.	
PG7	**O feleditse leng go nna rrangwana yo o tlang a tshela?**	WRITE Mo&Yr here→	_____ /_____<br>**MON / YYYY**
PG8	**O na le bana ba le kae ba ba tlhokafetseng ba na le dingwaga tse di magareng ga 0 le 5?**	WRITE number here→<br>97 if never fathered a child	
PG9	**O na le <u>bana</u> ba le kae ba eleng <u>ba gago ka tsalo</u>? Seo se raya bana ba eleng ba gago ka sebele.**	WRITE number here→	
PG10	**A o kgotsofaletse palo ya bana ba o nang le bone ga jaana?**	1 = Ga ke a kgotsofala gotlhegotlhe<br>2 = Ga ke a kgotsofala go sekae<br>3= Ga ke na maikutlo / ke fa gare<br>4 = Ke kgotsofetse<br>5 = Ke kgotsofetse thata	
PG11	**O ka rata go nna le palo e e kae ya bana?**	WRITE number here→	
PG12	**Bana ba gago ba na le bommaabone ba le kae ba ba farologaneng ba tlholego?**	WRITE number here→<br>97 if never fathered a child	
PG13	**Mo dikgweding tse 3 tse di fetileng, a wena kgotsa molekane wa gago o ile a/wa batla go ithwala? [Read options if needed]**	1 = Ke ne ke batla, mme motho yo ke ratanang le ene o ne a sa batle<br>2 = Ke ne ke sa batle, mme motho yo ke ratanang le ene o ne a batla<br>3 = Re ne re batla roobedi<br>4 = Re ne re sa batle roobedi	

Date: ___ ___ /___ ___ ___ / ___ ___ ___ ___
DD / MON / Y Y Y Y **Put 5 digit PIN here → ___________________**

PG14	**A wena kgotsa motho yo o ratanang le ene o batla go ithwala mo ngwageng e e tlang? [Read out options]**	1 = Ke a batla, mme motho yo ke ratanang le ene ga a batle<br>2 = Ga ke batle, mme motho yo ke ratanang le ene o a batla<br>3 = Re a batla roobedi<br>4 = Ga re batle roobedi.	
PG15	**Ke dingwaga dife tse di gaisang tsotlhe tse mosadi a tshwanetseng go itlhwala lekgetlo la ntlha ka tsone?**	WRITE age here→	
PG16	**Ke dingwaga dife tse di gaisang tsotlhe tse monna a tshwanetseng go nna le ngwana ka tsone?**	WRITE age here→	
PG17	**O ne o na le dingwaga tse kae fa o robalana lekgetlo la ntlha (o dirisa bosadi kgotsa sebono)?**	WRITE age here→	

Jaanong ke tlile go go botsa ka dilo tse o di dumelang. Batho ba na le dilo di le dintsi tse di farologaneng tse ba di dumelang. Sa ntlha, ke batla go utlwa gore o dumelang ka dikhontomo. Ke tlile go go balela polelo fano, ke kopa gore o mpolelele gore o dumalana kgotsa ga o dumalane go le kana kang le polelo eno o dirisa nommoro e e leng mo karateng eno. Gakologelwa gore ga go na dikarabo tse di siameng kgotsa tse di phoso. Ke kopa gore o mpolelele kafa o ikutlwang ka teng.

[Hand out card; explain how to use it]

		Ga ke dumalane gotlhelele	Ga ke dumalane	Ke a dumalana	Ga ke dumalane Ke a dumalana
CU1	**Ke kgona go tlhotlheletsa molekane wa me go dirisa dikhontomo le fa a sa batle go di dirisa.**	**1**	**2**	**3**	**4**
CU2	**Ke ne nka gakologelwa go dirisa khondomo le eleng morago ga ke ntse ke nwa.**	**1**	**2**	**3**	**4**
CU3	**Ke ne nka kgaotsa ke bo ke tsenya khondomo ka bonna kgotsa gore motho yo ke ratanang le ene a e tsenye le eleng fa re tsogetswe thata roobedi.**	**1**	**2**	**3**	**4**
CU4	**Nka gana go robalana fa go sena khondomo.**	**1**	**2**	**3**	**4**
CU5	**Fa ke ne nka tshitshinya gore re dirise dikhondomo le motho yo ke ratanang le ene yo ke ntseng ke sa dirise dikhondomo le ene, ke ne nka tshaba gore o ne a ka nkgana.**	**1**	**2**	**3**	**4**
CU6	**Fa ke ne nka tshitshinya gore re dirise dikhondomo le motho yo moswa go ratana le ene o ne a ka akanya gore ke na le bolwetse bongwe jo bo tshelanwang ka go robalana.**	**1**	**2**	**3**	**4**
CU7	**Fa ke ne nka tshitshinya gore re dirise dikhondomo le motho yo moswa go ratana le ene o ne a ka akanya gore ke ne ke ithaya ke re ba na le bolwetse bongwe jo bo tshelanwang ka go robalana.**	**1**	**2**	**3**	**4**

77= ganne 88= ga ke itse (if not already listed) 97= ga e maleba 99= tshedimosetso e e tlhaelang

Date: ___ ___ /___ ___ ___ / ___ ___ ___ ___
DD / MON / Y Y Y Y **Put 5 digit PIN here → ___________________**

Jaanong ke tlile go go botsa dipotso di sekae kaga dilo tse o di dumelang tse di amanang le tsalo. Ke kopa gore o mpolelele gore o dumalana kgotsa ga o dumalane go le kana kang le dipolelo tse di latelang. [Repeat instructions and reminders]

	**[Provide response card]**	**Ga ke dumalane gotlhelele**	**Ga ke dumalane**	**Ke a dumalana**	**Ga ke dumalane Ke a dumalana**
PB1	**Mosetsana o tshwanetse go ithwala go bontsha gore o kgona go belega.**	1	2	3	4
PB2	**Monna o tla batla go nyala mosetsana fela fa e le gore ke ene o mo ithwadisitseng mo nakong e e fetileng.**	1	2	3	4
PB3	**Mosetsana ga se mosadi kwantle ga fa a kile a nna le ngwana.**	1	2	3	4
PB4	**Mosimane ga se monna go fitlhela a sena go nna le ngwana le mosadi.**	1	2	3	4
PB5	**Ke matlhabisaditlhong mo lelapeng fa mosetsana a ithwala pele ga a nyalwa.**	1	2	3	4
PB6	**Monna o tla batla go nyala mosetsana fela fa a mo tsaletse ngwana/bana.**	1	2	3	4
PB7	**Batsadi ba bona selekanyo se se kwa tlase sa bogadi fa morwadiabone a ne a ithwele mme a nnile le ngwana.**	1	2	3	4
PB8	**Batho ba sesadi ba a ithwala gore ba bone madi a puso a go tlhokomela bana.**	1	2	3	4
PB9	**Mosadi o dumela gore o tshwanela go ithwala go bontsha gore ke mosadi wa mmatota.**	1	2	3	4
PB10	**Ditsala di tlhotlheletsana go ithwala.**	1	2	3	4
PB11	**Go ithwala ke letshwao la gore o mosadi.**	1	2	3	4
PB12	**Banna ba dumela gore mosadi o tshwanela go ithwala go bontsha gore ke mosadi wa mmatota.**	1	2	3	4

77= ganne 88= ga ke itse (if not already listed) 97= ga e maleba 99= tshedimosetso e e tlhaelang

Date: ___ ___ /___ ___ ___ / ___ ___ ___ ___
DD / MON / Y Y Y Y Put 5 digit PIN here → ________________

Jaanong go latela dipotso di sekae kaga dilo tse o di dumelang kaga dikamano magareng ga banna le basadi.

	**[Provide response card]**	**Ga ke dumalane Ga ke dumalane**	**Ga ke dumalane**	**Ke a dumalana**	**Ke Dumalana ka Botlalo**
R1	Banna ba na le batho ba bantsi ba ba ratanang le bone ka gonne ba ntse jalo ka tlhago	1	2	3	4
R2	Banna ba na le batho ba ba ratanang le bone gore ba bone maikatlapelo go ka kgotsofatsa balekane ba bone ba ba robalanang le bone gantsi	1	2	3	4
R3	Matsatsing ano basadi ba re ba tshwanela go nna le balekane ba ba fetang a le mongwe ba ba robalanang le bone	1	2	3	4
R4	Banna ba tlhabisiwa ditlhong ke basadi ba bone mme ba batla batho ba baswa ba ba ratanang le bone ba ba ka tsamayang le bone go ba bontsha ditsala tsa bone	1	2	3	4
R5	Fa banna ba sena batho ba ba ratanang le bone ditsala tsa bone di a ba tshega	1	2	3	4
R6	Basadi ba ba ikemetseng sentle ka ditšhelete ga ba batle go ikgolega ka motho a le mongwe yo ba ratanang le ene	1	2	3	4
R7	Malapa a a nang le baswa ba ba berekang ga a batle ba nyala/nyalwa ka gonne ba tshaba go latlhegelwa ke madi a lotseno a bone	1	2	3	4
R8	Gantsi banna ba pateletsa basadi ka bokukuntshwane gore ba robalane le bone le eleng fa ba sa batle go robalana le bone	1	2	3	4
**Go siame fa monna a betsa mosadi wa gagwe kgotsa a mo itaya fa a...**					
PWR1	…ya gongwe a sa mmolelela.	1	2	3	4
PWR2	...a sa tlhokomele bana.	1	2	3	4
PWR3	…ngangisana le ene.	1	2	3	4
PWR4	... a gana go robalana le ene.	1	2	3	4
PWR5	...a fisa dijo.	1	2	3	4

Sa bofelo, go latela dipotso di sekae kaga ditsela tsa go thibela mogare wa HIV.

		**CODE**<br>0= Nnyaa<br>1= Ee<br>2= Ga ke itse sentle
K1	A ga jaana go na le moento o o dirang gore batho ba seka ba tsenwa ke mogare wa HIV?	
K2	A ga jaana go na le moento o o fokotsang lebelo la bolwetse morago ga batho ba sena go tsenwa ke mogare wa HIV?	
K3	A ga jaana go na le jele nngwe e basadi ba ka e itshasang mo bosading mme e bo e dira gore ba seka ba tsenwa ke mogare wa HIV ka nako ya thobalano?	

K4	Fa monna a rupisitswe mo bonneng ke ngaka, a go na le kgonagalo e e kwa tlase ya gore a tsenwe ke mogare wa HIV?	

Pampiri eno ya dipotso e felela fano. Re lebogela nako e o e iphileng go araba dipotso tseno.

TIMEEND	**Time Interview Ended**	HH:MM using 24 hour clock	___ ___ : ___ ___

[Refer as needed. Correct knowledge as needed using text below]:

- Ke batla go netefatsa gore o itse gore ga jaana ga go na moento o o emisang kgotsa o o ngotlang lebelo la mogare wa HIV.
- Ga jaana ga go na jele e basadi ba ka e itshasang mo bosading mme go dira gore ba seka ba tsenwa ke mogare wa HIV ka nako ya thobalano.
- Go dirwa diteko ka meento le dijele mo Aforika Borwa go bona gore a di a dira. Mme ga jaana ga re na moento kgotsa jele e e dirang.
- Banna ba ba rupisitsweng mo bonneng ke ngaka ba na le kgonagalo e e kwa tlase ya go tsenwa ke mogare wa HIV. Nka nna ka go fa tshedimosetso ya go ikgolaganya le ngaka e e rupisang batho.

Date: ___ ___ /___ ___ ___ / ___ ___ ___ ___
DD / MON / Y Y Y Y **Put 5 digit PIN here → ____________________**

INFORMATION TO BE COMPLETED BY INTERVIEWER AFTER INTERVIEW:

			CODE
I1	**Interviewer code for interviewer**		
I2	**In what language was the interview administered?**	0=Seesemane<br>1=Setswana<br>2=Seburu<br>3=Seesemane se se tlhakaneng le Setswana<br>4=Sexhosa<br>5=Sezulu<br>6=Tse dingwe	
I2o		If other specify→	

INFORMATION TO BE COMPLETED BY STUDY COORDINATOR OR DESIGNEE:

		**CODE**
**Is the participant eligible for IAVI Protocol B?**	0= Ineligible<br>1= Eligible<br>97= Not applicable	
