# Supplementary material for: HIV incidence and predictors of inconsistent condom use among adult men enrolled into an HIV vaccine preparedness study, Rustenburg, South Africa
Source: PLoS One. 2019 Apr 3;14(4):e0214786. doi: 10.1371/journal.pone.0214786 (PMC6447216; doi:10.1371/journal.pone.0214786)
Supplement: S4 File — (PDF) [file pone.0214786.s006.pdf]

Date: ___ ___ /___ ___ ___ / ___ ___ ___ ___
DD / MON / Y Y Y Y **Put 5 digit PIN here → _______________**

Start time: ________:_______
hh mm

AURUM CLINICAL ASQ

**Mo dikgweding di le 3 tse di fetileng...**			**CODE**
STI1	**…a go na le ngaka nngwe kgotsa mooki mongwe yo o kileng a go tlhatlhobela bolwetse bope fela jo bo tshelanwang ka thobalano (STI)?**	0= Nnyaa<br>1= Ee	
STI2	**...a o kile wa okelwa bolwetse jo bo tshelanwang ka thobalano (STI)?**	0= Nnyaa<br>1= Ee	
STI3<br>RIS14	**...a o setse o kile wa tswa seeledi sepe fela se se nkgang kgotsa se se sa tlwaelegang go tswa mo bosading kgotsa mo bonneng?**	0= Nnyaa<br>1= Ee	
STI4	**...a o kile wa utlwa botlhoko fa o ntsha motlhapo?**	0= Nnyaa<br>1= Ee	
STI5	**...a o kile wa nna le peine ka nako ya thobalano?**	0= Nnyaa<br>1= Ee	
STI6<br>RIS15	**...a o setse o kile wa nna le ntho mo bosading kgotsa mo bonneng?**	0= Nnyaa<br>1= Ee	
STI7	**IF FEMALE...a o kile wa nna le peine mo karolong e e kwa tlase mo mpeng?<br>Code=97 if male**	0= Nnyaa<br>1= Ee	
STI8	**IF FEMALE: ...a o kile wa tlhotlhonelwa kgotsa wa ikutlwa ekete o a fisa mo bosading?<br>Code=97 if male**	0= Nnyaa<br>1= Ee	

ASK OF MEN ONLY (if female code = 97)

**Jaanong go latela dipotso tse di leng kaga go rupisiwa mo bonneng jwa gago—eleng thulaganyo e go tlosiwang letlalo la molomo wa bonna jwa gago mo go yone.**			**CODE**
CIR1	**A o setse o kile wa rupisiwa mo bonneng ke ngaka kgotsa ke mooki?**	0= Nnyaa<br>1= Ee	
CIR2	**[If yes, circumcised by doctor/nurse]<br>O ne o na le dingwaga tse kae fa o ne o rupisiwa ke ngaka kgotsa mooki?**	Code age in years	
CIR3	**[If no, not medically circumcised]<br>A o ka rata go direlwa lekwalo la gore o ye go bona ngaka e e ka go rupisang?**	0= Nnyaa<br>1= Ee	
CIR4	**A o setse o kile wa rupisiwa mo bonneng kwa bogwera?**	0=Nnyaa<br>1=Ee	
CIR5	**[If circumcised in cultural practice]<br>O ne o na le dingwaga tse kae fa o rupisiwa kwa bogwera?**	Code age in years	

77= ganne 88= ga ke itse (if not already listed) 97= ga e maleba 99= tshedimosetso e e tlhaelang

Date: ___ ___ /___ ___ ___ / ___ ___ ___ ___
DD / MON / Y Y Y Y **Put 5 digit PIN here → ________________**

Jaanong ke tlile go go botsa ka dilo tse wena kgotsa motho yo o ratanang le ene lo ka tswang lo di dirisitse <u>mo dikgweding tse 3 tse di fetileng.</u> Fa e le gore o ne o na le motho a le mongwe yo o ratanang le ene mo dikgweding tse 3 tse di fetileng, ke kopa gore o akanye ka motho yoo fa o araba dipotso tseno. Fa e le gore o ne o na le batho ba ba fetang a le mongwe ba o ratanang le bone mo dikgweding tse tharo tse di fetileng, ke kopa gore o akanye ka motho yo o ratanang le ene yo o robalaneng le ene gantsi go feta botlhe.

	**Jaanong ke tlile go go balela lenaane. Ke kopa gore o mpolelele gore a wena kgotsa motho yo o ratanang le ene o dirisitse mokgwa ope fela mo go e e latelang mo dikgweding tse 3 tse di fetileng...**	**CODES**<br>0=Nnyaa<br>1=Ee<br>88=Ga Ke Itse<br>97= ga e maleba, ga ke a robalana le ope
C1	**go tlhabiwa ka dihoromone (depo)**	
C2	**dipilise tsa dihoromone tsa go thibela pelegi (pilise)**	
C3	**wa senya mpa (ka boomo)**	
	**Jaanong ke kopa gore o mpolelele gore a wena kgotsa motho yo o ratanang le ene o dirisitse mokgwa ope fela mo go e e latelang <u>gantsi go le kana kang</u> mo dikgweding tse tharo tse di fetileng...**	**CODES**<br>0=Ga ke ise ke dirise ope<br>1=Ka dinako tse dingwe<br>2= halofo ya nako<br>3= Gantsi thata<br>4=Mo e ka nnag ka nako yotlhe<br>5= Ruri ka dinako tsotlhe<br>97= ga e maleba, ga ke a robalana le ope
C4	**dikhondomo tsa banna**	
C5	**dikhondomo tsa basadi**	
C6	**O ile wa somola bonna jwa gago pele ga peo ya bonna e tswa, kgotsa wa tshololela peo ya bonna kwa ntle ga bosadi**	

	**A wena kgotsa molekane wa gago o kile a/wa …**	**CODES**<br>0=Nnyaa<br>1=Ee
C7	**go bofiwa ditšhupu tsa popelo (go opofadiwa ga basadi kgotsa go bofiwa ga ditšhupu tsa bone tsa popelo)**	
C8	**hysterectomy (karo ya go tlosa popelo ya mosadi)**	
C9	**vasectomy (go opofadiwa ga banna)**	

	**Jaanong ke tlile go go botsa gore o robalane gantsi go le kana kang mo bekeng e e fetileng, ke gore, mo malatsing a le 7 a a fetileng.**		**CODE**
LW1	**O robalane mo bosading gantsi go le kana kang mo malatsing a le 7 a a fetileng, go akaretsa le gompieno?**	Code number of sex acts →	
LW2	**A palo eno e o robalaneng ka yone mo bosading mo bekeng e le nngwe ke selekanyo sa ka tlwaelo mo go wena (palo ya ka gale mo go wena ya go robalana)?**	0= Nnyaa<br>1= Ee	
LW3	**O robalane makgetlo a le kae mo sebonong mo bekeng e e fetileng?**	Code	

77= ganne 88= ga ke itse (if not already listed) 97= ga e maleba 99= tshedimosetso e e tlhaelang

		number of sex acts →	
LW4	**A palo eno e o robalaneng ka yone mo sebonong mo bekeng e le nngwe ke selekanyo sa ka tlwaelo mo go wena (palo ya ka gale mo go wena ya go robalana)?**	0= Nnyaa<br>1= Ee	

Date: ___ ___ /___ ___ ___ / ___ ___ ___ ___
DD / MON / Y Y Y Y Put 5 digit PIN here → _______________

				CODE
**ASK THESE OF PEOPLE WHO SAID THEY HAVE PULLED OUT THE PENIS IN LAST 3 MOS**				
CIY1	**Ke mang yo o tlileng ka tlhaloganyo ya gore o somole bonna jwa gago?**		0= E ne e le tlhaloganyo ya me<br>1= E ne e le tlhaloganyo ya motho yo ke ratanang le ene<br>2= Ka bobedi<br>3= E ne e se tlhaloganyo ya ope wa rona - go itiragaletse fela<br>4= Tse dingwe	
CIY2	**If code other above Write reason for pulling out here →**			
CIY3	**A wena le motho yo o ratanang le ene lo ile lwa buisana pele ga lo robalana kgotsa ka nako e lo neng lo robalane ka yone malebang le gore o somole bonna jwa gago?**		0= Nnyaa<br>1= Ee	
CIY4	**IF YES – Mpolelela gore kgang eno e tlile jang, le gore ke eng se lo neng lo tlotla ka sone →**			
CIY5	**Ke ka ntlha yang wena kgotsa motho yo o ratanang le ene a ile a somola bonna jwa gagwe?**<br><br>**[DO NOT READ ANSWERS OUT ALOUD – LET PARTICPANT ANSWER FIRST. USE OTHER IF NECESSARY]**		0= Go thibela HIV<br>1= Go thibela malwetse a a tshelanwang ka thobalano (STI)<br>2= Go thibela go ithwala<br>3= Go thibela go ithwala le bolwetse<br>4= Ga ke itse - go itiragaletse fela<br>5= Ka ntlha ya mabaka a setso - tlhalosa fa tlase fano<br>6= Tse dingwe	
CIY6	**If coded as 'cultural' or other above write reason for pulling out here →**			
CIY7	**O na le nako e e kae o ratana le motho yono (yo o somotseng bonna fa o na le ene?)**		Code in months for all total time together (take out times not together).<br>Code 0 if one time partner →	
	**Lekgetlo la bofelo le o somotseng bonna ka lone, ke kopa gore o mpolelele gore ke eng se sengwe se o neng o se dirisa ka yone nako eo...**			**CODES**<br>0=Nnyaa<br>1=Ee
CIY8	**...go tlhabiwa ka dihoromone (depo)**			
CIY9	**...dipilise tsa dihoromone tsa go thibela pelegi (pilise)**			
CIY10	**...dikhondomo tsa banna**			
CIY11	**...dikhondomo tsa basadi**			
CIY12	**...setso**			
CIY13	**Fa o rile setso, tlhalosa →**			

77= ganne 88= ga ke itse (if not already listed) 97= ga e maleba 99= tshedimosetso e e tlhaelang

Date: ___ ___ /___ ___ ___ / ___ ___ ___ ___
DD / MON / Y Y Y Y **Put 5 digit PIN here → _______________**

CIY14	**...tse dingwe? Tlhalosa→**		
CIY15	**A seno ke selo sa ka gale se o tlwaetseng go se dirisa fa o somola bonna?**		

**ASK THESE OF PEOPLE WHO SAID THEY HAVE NOT PULLED OUT THE PENIS IN LAST 3 MOS**			**CODE**
CIN1	**Ke ka ntlha yang o sa rate? Write reason for not using pulling out penis here →**		
CIN2	**A o setse o kile wa dirisa mokgwa wa go somola bonna?**	0= Nnyaa<br>1= Ee	
CIN3	**[IF YES TO EVER USE] Mpolelela gore kgang eno e tlile jang, le gore ke eng se lo neng lo tlotla ka sone**		
CIN4	**[IF YES TO EVER USE]<br>Ke ka ntlha yang wena kgotsa motho yo o ratanang le ene a ile a somola bonna jwa gagwe?<br><br>[DO NOT READ ANSWERS OUT LOUD – LET PARTICPANT ANSWER FIRST]**	0= Go thibela HIV<br>1= Go thibela malwetse a a tshelanwang ka thobalano (STI)<br>2= Go thibela go ithwala<br>3= Go thibela go ithwala le bolwetse<br>4= Ga ke itse - go itiragaletse fela<br>5= Tse dingwe	
CIN5	**[If code other above write reason for pulling out here] →**		
CIN6	**O ne o na le nako e e kae o ratana le motho yono (yo o somotseng bonna fa o na le ene)?**	Code in months for all total time together (take out times not together).<br>Code 0 if one time partner. →	
CIN7	**O ne o na le dingwaga tse kae lekgetlo la bofelo le o somotseng bonna ka lone?**	Code age here →	

77= ganne 88= ga ke itse (if not already listed) 97= ga e maleba 99= tshedimosetso e e tlhaelang

Date: ___ ___ /___ ___ ___ / ___ ___ ___ ___
DD / MON / Y Y Y Y   Put 5 digit PIN here → _______________

ASK THIS PAGE TO ALL PARTICIPANTS – NO MATTER WHAT THEIR PRACTICE IS – THERE ARE NO RIGHT OR WRONG ANSWERS HERE – THESE MEASURE PARTICIPANT BELIEFS –NOT PRACTICES

Jaanong ke rata go utlwa ka dilo tse o di dumelang malebang le go somola bonna pele ga peo ya bonna e tswa, kgotsa go tsholololela peo ya bonna kwa ntle. [Repeat instructions and reminders]

	**[Provide response card]**	**Ga ke dumalane gotlhelele**	**Ga ke dumalane**	**Ke a dumalana**	**Ga ke dumalane Ke a dumalana**
WDMP	Go nnile le dinako tse ke neng ke batla go somola bonna pele ga peo ya bonna e tswa fa ke ne ke na le (ba)motho yo ke ratanang segolo le ene	**1**	**2**	**3**	**4**
WDCP	Go nnile le dinako tse ke neng ke batla go somola bonna pele ga peo ya bonna e tswa fa ke ne ke na le (ba)motho yo ke ratanang go tsamaisa nako le ene	**1**	**2**	**3**	**4**
WD1	Ke kgona go tlhotlheletsa molekane wa me go somola bonna jwa gagwe (go tsholololela peo kwa ntle) le eleng fa a sa batle.	**1**	**2**	**3**	**4**
WD2	Ke ne nka gakologelwa go somola bonna pele le eleng morago ga ke ntse ke nwa bojalwa.	**1**	**2**	**3**	**4**
WD3	Ke ne nka netefatsa gore ke somole bonna (ke tshololele peo ya bonna kwa ntle) le eleng fa re tsogetswe thata roobedi.	**1**	**2**	**3**	**4**
WD4	Nka gana go robalana fa go sena sebaka sa go somola bonna.	**1**	**2**	**3**	**4**
WD5	Fa ke ne nka tshitshinya go somola bonna (go tsholololela peo ya bonna kwa ntle) fa ke na le motho yo ke ratanang le ene yo ke iseng ke tle ke dire seno le ene, ke ne nka boifa gore motho yoo yo ke ratanang le ene o ne a ka nkgana.	**1**	**2**	**3**	**4**
WD6	Fa ke ne nka tshitshinya go somola bonna motho yo ke ratanang le ene o ne a ka akanya gore ke na le bolwetse bongwe jo bo tshelanwang ka go robalana.	**1**	**2**	**3**	**4**
WD7	Fa ke ne nka tshitshinya go somola bonna motho yo moswa go ratana le ene o ne a ka akanya gore ke ithaya ke re ba na le bolwetse bongwe jo bo tshelanwang ka go robalana.	**1**	**2**	**3**	**4**
WD8	Fa ke ne nka tshitshinya go somola bonna motho yo ke ratanang le ene o ne a ka akanya gore ga ke batle go ithwala/ ga ke batle go mo dira gore a ithwale.	**1**	**2**	**3**	**4**
WD9	Fa ke ne nka somola bonna pele ga peo ya bonna e tswa, motho yo ke ratanang le ene o ne a ka akanya gore ke leka go tila go <u>tsenwa</u> ke mogare wa HIV.	**1**	**2**	**3**	**4**
WD10	Fa ke ne nka somola bonna pele ga peo ya bonna e tswa, motho yo ke ratanang le ene o ne a ka akanya gore ke leka go tila go mo <u>tshela</u> mogare wa HIV.	**1**	**2**	**3**	**4**

77= ganne   88= ga ke itse (if not already listed)   97= ga e maleba 99= tshedimosetso e e tlhaelang

WD11	Fa ke ne nka somola bonna, motho yo ke ratanang le ene o ne a ka akanya gore ke palelwa ke go fetsa tiro.	**1**	**2**	**3**	**4**
WD12	Fa ke ne nka somola bonna, motho yo ke ratanang le ene one a ka nja ditshego	**1**	**2**	**3**	**4**
WD13	Fa ke ne nka somola bonna, motho yo ke ratanang le ene o ne a ka akanya gore ke monna yo o maatla	**1**	**2**	**3**	**4**
WD14	Fa ke ne nka somola bonna, motho yo ke ratanang le ene o ne a ka akanya gore ke kgona go ikgapha tota.	**1**	**2**	**3**	**4**
WD15	Fa ke ne nka somola bonna, motho yo ke ratanang le ene o ne a ka akanya gore ga ke itse go robalana.	**1**	**2**	**3**	**4**

Date: ___ ___ /___ ___ ___ / ___ ___ ___ ___
DD / MON / Y Y Y Y **Put 5 digit PIN here →** _______________

			CODE
RIS16	**Mo dikgweding tse 3 tse di fetileng, ke batho ba le kae ba o ratanang le bone ba e neng e le batho ba senna?**	Enter no. of male partners →	
RISMCMFRQ	**Mo dikgweding tse 3 tse di fetileng, o dirisitse dikhondomo kgapetsakgapetsa go le kana kang fa o na le batho ba senna ba o ratanang le bone?**	0= Ga nke ke e dirisa<br>1= Ka dinako tse dingwe (kafa tlase ga halofo ya nako)<br>2= Kgapetsakgapetsa (go feta halofo ya nako)<br>3= Ka metlha<br>4= Ga ke a mmotsa (go ne go sena batho ba senna ba a ratanang le bone)	
RIS16a	**Mo bathong bano ba o ratanang le bone ba ba____ke ba le kae mo go bone ba e neng e le batho ba baswa go ratana le bone, ke gore, motho yo o neng o ise o ke o robalane le ene pele?**	Enter no. of new male partners →	
RIS16b	**O dirisitse dikhontomo gantsi go le kana kang fa o ne o robalana le batho bano ba baswa go ratana le bone ba ba____?**	0= Ga ke ise ke dirise ope<br>1= Ka dinako tse dingwe (kafa tlase ga halofo ya nako)<br>2= Kgapetsakgapetsa (go feta halofo ya nako)<br>3= Ka metlha<br>4= Ga ke a mmotsa (ga a na batho ba senna ba baswa go ratana le bone)	
RIS17	**Mo dikgweding tse tharo tse di fetileng, ke batho ba le kae ba o ratanang le bone ba e neng e le batho ba sesadi?**	Enter no. of female partners →	
RISMCFFRQ	**Mo dikgweding tse tharo tse di fetileng, o dirisitse dikhondomo kgapetsakgapetsa go le kana kang fa o na le batho ba sesadi ba o ratanang le bone?**	0= Ga nke ke e dirisa<br>1= Ka dinako tse dingwe (kafa tlase ga halofo ya nako)<br>2= Kgapetsakgapetsa (go feta halofo ya nako)<br>3= Ka metlha<br>4= Ga ke a mmotsa (go ne go sena batho ba sesadi ba a ratanang le bone)	
RIS17a	**Mo bathong bano ba o ratanang le bone ba ba____ke ba le ba kae mo go bone ba e neng e le batho ba baswa go ratana le bone, ke gore, motho yo o neng o ise o ke o robalane le ene pele?**	Enter no. of new female partners →	
RIS17b	**O dirisitse dikhontomo gantsi go le kana kang fa o ne o robalana le batho bano ba sesadi ba baswa go ratana le bone ba ba____?**	0= Ga ke ise ke dirise ope<br>1= Ka dinako tse dingwe (kafa tlase ga halofo ya nako)<br>2= Kgapetsakgapetsa (go feta halofo ya nako)<br>3= Ka metlha<br>4= Ga ke a mmotsa (ga a na batho ba sesadi ba baswa go ratana le bone)	
RIS28	**Ke batho ba le kae bano banna senna le ba sesadi ba o ratanang le bone ba ba _____ba ba neng ba na le mogare wa HIV?**	0= Nnyaa<br>1= Ee → if yes, go to next Q<br>88= Ga ke itse	
PAR2	**A (ba)motho yono yo o ratanang le ene yo o nang le mogare wa HIV o dirisitse di-ARV (melemo ya HIV) mo dikgweding tse 3 tse di fetileng?**	0= Ga a dirisa epe<br>1= O dirisitse dingwe<br>2= Tsotlhe<br>88= Ga ke itse	

77= ganne 88= ga ke itse (if not already listed) 97= ga e maleba 99= tshedimosetso e e tlhaelang

RIS28b	**O dirisitse dikhondomo gantsi go le kana kang fa o ne o robalana le batho bano ba o ratanang le bone ba ba nang le mogare wa HIV?**	0= Ga ke ise ke dirise ope<br>1= Ka dinako tse dingwe (kafa tlase ga halofo ya nako)<br>2= Kgapetsakgapetsa (go feta halofo ya nako)<br>3= Ka metlha<br>4= Ga ke a mmotsa (ga a na batho ba a ratanang le bone ba ba nang le HIV)	
RIS29	**Mo bathong bano ba o robalanang le bone ba ba ____ ke ba le kae mo go bone ba o *amogetseng* madi, dimpho kgotsa thuso nngwe gore o robalane le bone?** (fa ke re dimpho kgotsa thuso ke raya go fiwa tuelo, ka sepalangwa, ka dijo, ka metsotso ya founu, ka diaparo, ka madi kgotsa ka sengwe le sengwe se sele, mme tuelo eno e ke nna ya tswa e sa dirwa ka nako ya thobalano.)	Code number →	
RIS210	**Mo bathong bano ba o robalanang le bone ba ba ____ ke ba le kae mo go bone ba o *fileng* madi, dimpho kgotsa thuso nngwe gore o robalane le bone?** (fa ke re dimpho kgotsa thuso ke raya go fiwa tuelo, ka sepalangwa, ka dijo, ka metsotso ya founu, ka diaparo, ka madi kgotsa ka sengwe le sengwe se sele, mme tuelo eno e ke nna ya tswa e sa dirwa ka nako ya thobalano.)	Code number →	
RIS211	**Mo dikgweding tse 3 tse di fetileng, a o setse o kile wa patelediwa ke mongwe gore o robalane le ene o sa batle?**	0= Nnyaa<br>1= Ee → refer to supervisor for f/up	
RIS212	**Mo dikgweding tse 3 tse di fetileng, a o ile wa tsaya loeto kgotsa wa se robale gae kgapetsa-kgapetsa?** (beke ya masigo a palogare e e fetang 3 ka beke)	0= Nnyaa<br>1= Ee	
RIS213	**Mo dikgweding tse 3 tse di fetileng a o ile wa robalana le batho ka setlhopha, ke gore, a o ile wa robalana le batho ba ba fetang a le mongwe ka nako e le nngwe?**	0= Nnyaa<br>1= Ee, basadi fela<br>2= ee, banna fela<br>3= Ee, banna le basadi<br>4= Ga ke a e botsa	
RIS214	**Mo dikgweding tse 3 tse di fetileng, a o ile wa robalana ka sebono, ke gore, moo motho yo o robalanang le ene a neng a tsentse bonna jwa gagwe mo sebonong sa gago?**	0= Nnyaa<br>1= Ee<br>2= Ga ke a e botsa	
RIS214a	**O ile wa dirisa dikhontomo gantsi go le kana kang fa o robalana ka sebono, ke gore, moo molekane wa gago yo o robalanang le ene a neng a tsentse bonna jwa gagwe mo sebonong sa gago?**	0= Ga ke ise ke dirise ope<br>1= Ka dinako tse dingwe (kafa tlase ga halofo ya nako)<br>2= Kgapetsakgapetsa (go feta halofo ya nako)<br>3= Ka metlha<br>4= Ga ke a e botsa (ga a robalana ka sebono)	
RIS215			

77= ganne 88= ga ke itse (if not already listed) 97= ga e maleba 99= tshedimosetso e e tlhaelang

	**MEN ONLY: Mo dikgweding tse 3 tse di fetileng, a o ile wa robalana ka sebono, ke gore, moo o neng o tsentse bonna jwa gago mo sebonong sa molekane wa gago yo o robalanang le?**	0= Nnyaa<br>1= Ee<br>2= Ga ke a e botsa	
RIS215	**MEN ONLY: O ile wa dirisa khondomo gantsi go le kana kang fa o robalana ka sebono, ke gore, moo o neng o tsentse bonna jwa gago mo sebonong sa molekane wa gago yo o robalanang le ene?**	0= Ga ke ise ke dirise ope<br>1= Ka dinako tse dingwe (kafa tlase ga halofo ya nako)<br>2= Kgapetsakgapetsa (go feta halofo ya nako)<br>3= Ka metlha<br>4= Ga ke a e botsa (ga a robalana ka sebono)	

77= ganne 88= ga ke itse (if not already listed) 97= ga e maleba 99= tshedimosetso e e tlhaelang

Date: ___ ___ /___ ___ ___ / ___ ___ ___ ___

DD / MON / Y Y Y Y **Put 5 digit PIN here →** ________________

COLLECT FROM ALL: VITAL SIGNS (Nurse, enrolled nurse, doctor or clinician to take vitals)

			CODE
V0	**Who took these vitals?**	0=Enrolled Nurse<br>1= Professional Nurse<br>2= Ngaka<br>3= Tse dingwe	
V1BP<br>PEX11	**Blood pressure**	record mm/Hg→	_ _ _ / _ _ _
V2P<br>PEX12	**Pulse**	record beats per minute→	
VRR<br>PEX13	**Respiration Rate**	record breaths per minute →	_ _
VBT<br>PEX14	**Body Temperature**	record to 1 decimal place in degrees Celcius (XX.X) →	_ _ . _
V5 W<br>PEX15	**Weight**	record in kg→	_ _ _
V6H<br>PEX16	**Height**	record in cm→	_ _ _

TO BE COMPLETED BY THE NURSE/DOCTOR, BASED ON PROFESSIONAL JUDGEMENT

		YES	NO
ELI1 9	**Presence of any condition that would interfere with achieving the study objectives?**		

____
Staff code

End time: _______:______
hh mm

Letlha: ___ ___ /___ ___ ___ / ___ ___ ___ ___
LETSATSI / KGWEDI / NGWAGA

77= ganne 88= ga ke itse (if not already listed) 97= ga e maleba 99= tshedimosetso e e tlhaelang
