# Supplementary material for: HIV incidence and predictors of inconsistent condom use among adult men enrolled into an HIV vaccine preparedness study, Rustenburg, South Africa
Source: PLoS One. 2019 Apr 3;14(4):e0214786. doi: 10.1371/journal.pone.0214786 (PMC6447216; doi:10.1371/journal.pone.0214786)
Supplement: S5 File — (PDF) [file pone.0214786.s007.pdf]

Umhla: ___ ___ /___ ___ ___ / ___ ___ ___ ___
DD / MON / Y Y Y Y **Put 5 digit PIN here →** ________________

Start time: ________:______

Ikhweshine i-Aurum Screening Questionnaire

- **Enkosi ngokuvuma ukuthatha inxaxheba kolu phando lwethu.**

- **Sinomdla ekubeni sazi ngempilo yabantu. Eminye kule mibuzo inokungaphathi kakuhle, kodwa kubalulekile kakhulu ukuba sazi inyaniso ngoko kuqhubekayo phakathi kwabahlali. Akukho zimpendulo zilungileyo nezingalunganga. Nceda unyaniseke xa uphendula imibuzo.**

- **Ungangayiphenduli imibuzo nokuba yeyiphi na ukuba akufuni kuyiphendula – into ebalulekileyo kukuba uzive uphatheke kakuhle kukuyiphendula ngokunyanisekileyo.**

- **Iimpendulo zakho kule mibuzo ziya kusixelela ngoluntu oluya kusinceda kwiiprojekthi zophando zexesha elizayo.**

- **Zonke iimpendulo zakho ziya kugcinwa ziyimfihlo. Ukuthi yimfihlo kuthetha ukuba asiyi kuzixela iimpendulo zakho nakubani na ongaphandle kweli qela lophando.**

Umhla: ___ ___ /___ ___ ___ / ___ ___ ___ ___
DD / MON / Y Y Y Y **Put 5 digit PIN here →** __________________

Umncedisi wezophando			**CODE**
D1	**Weva njani okokuqala ngqa ngolu phando? Nceda ukhethe impendulo ibenye kweli khadi.**	1= Eklinikhi<br>2= Ndachazelwa ngabamelwane bam<br>3= NgeVCT emobhayili<br>4= Kwintlanganiso yoluntu okanye ngaphandle phakathi kwabahlali<br>5= Ndeva ngayo kubahlobo bam/kusapho lwam<br>6= Kwiqabane lam<br>7= Esikolweni<br>8= Kwindawo ekuncedwa kuyo abantu<br>9= Ethaveni/eshibhini/kwindawo ekuselwa kuyo utywala<br>10= Enye (zixele)	
D2	If code 10 above,,write where they heard about Aurum from here →		
D3	**Uneminyaka emingaphi ubudala?**	Code in years→	
D4	**Uthini umhla wokuzalwa kwakho?**	Write in date	___ ___ / ___ ___ ___ / ___ ___ ___ ___<br>USUKU / INYANGA / UNYAKA
D5	**Uhlala kweyiphi indawo eRustenburg?** Write area here→		
D6	**Sithini isini sakho? Oko kukuthi uyindoda, ulibhinqa, okanye ungumntu ongazichazi njengokuba yindoda okanye ibhinqa?**	0= Iyindoda<br>1= Ibhinqa<br>2= Umntu onxiba iimpahla zesinye isini /ozelwe eseso sini kodwa azithathe njengesinye isini	
D7	**Ungayichaza njani imeko yakho yezengqesho? Nceda ukhethe kweli khadi.**	0= Akuqeshwanga ukhangela umsebenzi<br>1= Akuqeshwanga – akukhangeli msebenzi<br>2= Akunako ukusebenza –ufumana igranti karhulumente<br>3= Umfundi<br>4= Uyazisebenzela - okwethutyana nganeno kwiiyure eziyi- 40 ngeveki<br>5= Uziqeshile ngokwakho –isigxina- iiyure eziyi-40 okanye ngaphezulu ngeveki<br>6= Uqeshiwe, ngaphantsi kweeyure eziyi-40 ngeveki<br>7= Uqeshiwe, isigxina (iiyure eziyi-40 okanye ngaphezulu)<br>8= Enye	
D7o	If code 8 above, write employment situation→		
D8	**Ungayichaza njani imeko yakho yezemali yosapho lwakho?**	1= Imali engonelanga yokuthenga izinto ezingundoqo ezifana nokutya, iimpahla<br>2= Ukuba nemali yokutya neempahla kodwa ziyashota ezinye izinto ezininzi<br>3= Sinazo izinto ezingundoqo kodwa asinamali yaneleyo yezinto ezibiza phezulu<br>4= Sinemali yokonga okanye ukuthenga izinto ezibiza phezulu<br>5= Enye	
D8o	If code 5 above, write family situation →		

Umhla: ___ ___ /___ ___ ___ / ___ ___ ___ ___
DD / MON / Y Y Y Y **Put 5 digit PIN here →** ___________________

D9	**Xa kuthelekiswa nezinye iintsapho ubungathi usapho lwakho luneentsiba noko, luyafana nje okanye luhlupheke kakhulu kunezinye iintsapho?**	0= Luhluphekile kunezinye<br>1= Luyafana nje nezinye<br>2= Luneentsiba kunezinye	
D10	**Xa kuthelekiswa nabanye oogxa bakho ubungathi usapho lwakho luneentsiba noko, luyafana nje okanye luhlupheke kakhulu?**	0= Luhluphekile kunezinye<br>1= Luyafana nje nezinye<br>2= Luneentsiba kunezinye	

			**CODE**
D11<br>DEM2 8	**Ubuyintoni umthombo wakho ongundoqo wengeniso kwezi nyanga ziyi-12 zidlulileyo? (code up to 2 choices: first choice here, second choice in next question)**	1= Usapho<br>2= Ukuqeshwa okufanelekileyo<br>3= Umsebenzi wethutyana/ukungxungxa<br>4= Iqabane, eliyindoda, eliyintombazana<br>5= Ukuziqesha<br>6= Azikho<br>7= Enye	
D12<br>DEM2 8o	If code 7 above Write primary source of income here→		
D13<br>DEM2 8	**Ukho omnye umthombo wengeniso?**	1= Usapho<br>2= Ukuqeshwa okufanelekileyo<br>3= Umsebenzi wethutyana/ukungxungxa<br>4= Iqabane, eliyindoda, eliyintombazana<br>5= Ukuziqesha<br>6= Azikho<br>7= Enye	
D14<br>DEM28o	If code 7 above Write primary source of income here→		
D15<br>DEM2 9	**Ingaba kungokunje unabaxhomekeke kuwe obanonophelayo (ngokwemali)?**	0= Hayi<br>1=Ewe	
D16	**Ungayichaza njani imeko yakho yezobudlelwane bezothando?**<br><br>**[Pick from card]**	1= Utshatile (umtshato wakwamantyi /owesintu /owenkolo)<br>2= Akutshatanga kodwa nihlala kunye<br>3= Akutshatanga kodwa uneqabane ekudala uthandana nalo<br>4= Akutshatanga kodwa uneqabane ekudala uthandana nalo neqabane (namaqabane) lamathuba athile<br>5= Akutshatanga kodwa uneqabane (namaqabane) lamathuba athile<br>6= Akutshatanga yaye akunaqabane<br>7= Enye	
D16o	**If code 7 above**	Write relationship status here→	
D17	**Ingaba iqabane lakho lihlala nawe?**	0= Hayi<br>1=Ewe	
D18	**[If has only one partner of any kind]**<br>**Unethuba elingakanani uneli qabane?**	Ikhowudi ngeenyanga ngawo onke amaxesha nikunye. Ukuthatha ixesha niphume kodwa ningekho kunye. Code 0 if one time partner →	
D19	**[If any partners]**	1= Ngaphantsi kunakanye ngonyaka	

	**Kule nyanga iphelileyo, ulibone iqabane (amaqabane) lakho amaxa amangaphi?**	2= Malunga nakanye ngenyanga<br>3= Malunga naka-2 ukuya ka-3 ngenyanga<br>4= Kanye ngeveki<br>5= Ngaphantsi kunakanye ngeveki	
D20	**Nceda undixelele ukuba zeziphi iinkcazelo kwezi ezichaza ngokugqibeleleyo imeko yakho yezesini?**	0= Ungothandana nesini esahlukileyo kwesakho<br>1= Omntu othandana nesini esifana nesakhe<br>2= Uthandana nabesini esifana nesakho nabesahlukileyo kwangokunjalo<br>3= Umntu othandana nesinye isini kodwa ngamanye amaxesha abelane ngesondo nomntu wesini esifana nesakhe<br>4= Umntu othandana nesini esifana nesakhe kodwa ngamanye amaxesha abelane ngesondo nabantu abanesini esingafaniyo nesakhe<br>5= Andikhethe sini sitheni, ndilala nabani na endimthandayo	

77= ndalile 88= andazi (ukuba ayisele idwelisiwe) 97= ayifanelekanga 99= idatha ayikho

Umhla: ___ ___ /___ ___ ___ / ___ ___ ___ ___
DD / MON / Y Y Y Y **Put 5 digit PIN here →** ___________________

Ngoku yimbuzo emalunga nawe

			CODE
DEM11	**Uloluphi uhlanga?**	1= ONtsundu<br>2= OMhlophe<br>3= Um-Ashiya<br>4= Olunye (cacisa apha ngezantsi)	
DEM11o	Write in other here→		
DEM12	**Leliphi iqela lakho lohlanga?**	Write in name here →	
DEM 13	**Wazalelwa kweliphi ilizwe?**	Write in name here →	
**Mingaphi iminyaka eyitotali yesikolo oyigqibileyo kwikhategori nganye?**			
DEM14a	**a. EyasePrayimari**	Code number of years →	
DEM14b	**b. EyaseSekondari**	Code number of years →	
DEM 14c	**c. Eyasemva kweyaseSekondari**	Code number of years →	
DEM14d	**d. Iminyaka egqityiweyo yesikolo/yoqeqesho**	Code number of years →	
DEM14e	**If has other school/apprenticeship, specify type here→**		
DEM15	**Yeyiphi inkolo yakho?**	1= UmKhatolika<br>2= UmProtestanti<br>3= Omnye umKrestu<br>4= UmSilamsi<br>5= Ayikho<br>6= Enye	
DEM15o	If above is other specify other religion here→		
DEM16	**Mingaphi iminyaka ohleli ngayo kule ndawo**	Code number of years; code= 00 if less than 1 year →	
DEM17	**Ithini imeko yakho yangoku malunga nomtshato?**	1= Akutshatanga<br>2= Uwuqhawule umtshato/wahlukene<br>3= Utshatile, unenkosikazi enye<br>4= Utshatile, unamakhosikazi amaninzi<br>4= Ungumhlolo/umhlolokazi	

77= ndalile 88= andazi (ukuba ayisele idwelisiwe) 97= ayifanelekanga 99= idatha ayikho

Umhla: ___ ___ /___ ___ ___ / ___ ___ ___ ___
DD / MON / Y Y Y Y **Put 5 digit PIN here →** ___________________

**Ngoku yeminye imibuzo ngezinto ongaba unazo okanye ongenazo. Le mibuzo ibhekiselele kwizinto <u>wena ngokwakho onazo</u> (hayi kwezo zisekhayeni lakho).**		
**Ingaba wena buqu…**		**USE THESE CODES 0= Hayi 1= Ewe**
PP1	**...usebenzisa iwotshi yesihlahla?**	
PP2	**...ijuwelari?** (umsesane, isihombiso somqala, amatsheyina enziwe ngegolide, ngeplatinum yesilivere njl.njl.)	
PP3	**…iselula esebenzayo?** (olunye uhlobo nokuba loluphi na)	
PP4	**…iselula esebenzayo enekhamera?**	
PP5	**….iselula esebenzayo ekhonekhtha ku-intanethi?** (Facebook, Twitter, MXIT)	
PP6	**…i-iPod esebenzayo /isixhobo somculo esisesakho /i-MP3 player?**	
PP7	**...ikhompyutha esebenzayo?** (i-laptop/i-desktop; naluphi uhlobo lwekhompyutha olusebenzayo= ewe)	
PP8	**…i-iPad esebenzayo**	
PP9	**…imoto yakho esebenzayo**	
PP10	**…iteksi esebenzayo okanye efana nesithuthi esifana naso esisetyenziselwa ukuthutha abakhweli**	
PP11	**…isithuthuthu esisebenzayo /isikuta?**	
PP12	**… indlu – eyodwa okanye edibene neyomnye umntu?**	
PP13	**… nawuphi umhlaba – owodwa okanye odibene nowomnye umntu?**	
PP14	**… itshekhi okanye i-akhawunti yokonga <u>ebhankini</u>?**	
PP15	**...ikhredithikhadi <u>yebhanki</u>?**	
PP16	**…imali-mboleko oyifumana kwivenkile ebolekisa ngemali?**	
PP17	**… naluphi na uhlobo lwekhredithikhadi yevenkile?**	

			**CODE**
PP18	**Uyonga kangakanani imali?**	0=Akongi<br>1= Wonga kanye ngonyaka (kubandakanya itshekhi ye-13)<br>2= Qho <u>kwiinyanga ezintandathu</u> okanye ezifana nezo<br>3= Qho <u>kwiinyanga ezimbalwa</u><br>4= Wonga <u>qho</u> ngenyanga	

77= ndalile 88= andazi (ukuba ayisele idwelisiwe) 97= ayifanelekanga 99= idatha ayikho

Umhla: ___ ___ /___ ___ ___ / ___ ___ ___ ___
DD / MON / Y Y Y Y **Put 5 digit PIN here →** ____________________

**Ngoku masithethe ngezinto ongaba unazo nezo ungenazo ekhayeni lakho. Xa ndibuza ngekhaya lakho ndithetha ngendawo apho uhlala khona ngoku –ingeyiyo ilali yakho apho uvela khona. Kule mibuzo ndithetha ngezinto ezisebenza kakuhle.**		
**Ingaba ikhaya lakho line…esebenza kakuhle.**		**USE THESE CODES 0= Hayi 1= Ewe**
HH1	**… IThivi?** (nokuba loluphi na uhlobo)	
HH2	**… iplasma okanye i-Thivi eyi-flat screen?**	
HH3	**…iDSTV, uMNET okanye u-TopTV, okanye enye isathelayithi yekhonekhshini yeThivi?**	
HH4	**…isistim yomculo nokunye okubukelwayo /isistim yesandi?**	
HH5	**…ifriji esebenzayo?**	
HH6	**…ifowuni yasendlini esebenzayo?**	
HH7	**…umbane osebenzayo?**	
HH8	**...itephu esebenzayo yamanzi endlwini?**	
HH9	**...itephu yamanzi esebenzayo eyadini yakho?**	

**Ndiza kukufundela uluhlu oluphathelele nokunika inkxaso abanye. Nceda undixelele ukuba ngamaxa amangaphi owenze ngawo ezi zinto kwezi nyanga zi-3 zidlulileyo.**		
	**Kwezi nyanga zi-3 zidlulileyo, ngamaxa amangaphi...**	**READ CODES OUT LOUD TO PARTICIPANT** 0=Azange 1= Amaxesha ambalwa 2=Qho ngenyanga okanye ngaphezulu
FS1	**…ukuthenga ukutya kosapho lwakho ukwenzela ikhaya?**	
FS2	**...ukuphisa ngokutya, imali okanye izinto ukuxhasa ikhaya losapho lwakho?**	
FS3	**...ukuphisa ngokutya, imali okanye izinto ukuxhasa amanye amalungu osapho (ngaphandle kwikhaya lakho elisondeleyo kuwe?**	
FS4	**…ukuphisa ngokutya, imali okanye izinto ukunika inkxaso abahlobo?**	
FS5	**…ukuphisa ngokutya, imali okanye izinto ukunceda ukuxhasa isithandwa**	

77= ndalile 88= andazi (ukuba ayisele idwelisiwe) 97= ayifanelekanga 99= idatha ayikho

	**(esingesiso inxenye yekhaya lakho ngokwakoku kungentla apha)?**	
FS6	**…ukuphisa ngokutya, imali okanye izinto ukunceda ukuxhasa umntu <u>omfunayo</u> abe sisithandwa sakho?**	

ASK OFALL—REGARDLESS OF ANY ANSWERS GIVEN ABOVE ABOUT EMPLOYEMENT

Ngoku ndingathanda ukukubuza eminye imibuzo ngemeko yomsebenzi wakho. Ungangayiphenduli nayiphi na imibuzo ongafuni kuyiphendula. Akukho zimpendulo zichanekileyo nezingachanekanga kule mibuzo. Nceda unyaniseke xa uphendulayo. Ngokufanayo nobalo-bantu, siyayibuza kuba sifuna ukwazi ngcono ngabahlali okanye uluntu.

E1	**Uyintoni umsebenzi wakho? Oko kukuthi wenza oluphi uhlobo lomsebenzi?**	Bhala apha umsebenzi uze uchaze kangangoko unako. If no job write not working	
E2	**Zingaphi iiyure, kubandakanywa nama-ova, othe wawasebenza kwezi <u>ntsuku ziyi-7 zidlulileyo</u>?**	Code number of hours worked (if did not work, code =0)→	**CODE**
E3	**Zingaphi iiyure <u>ngeveki</u>, kuqukwa nama-ova, <u>oqhele</u> ukuwasebenza?**	Code number of hours worked (if did not work, code =0)→	
E4	**Ingaba ufuna ukusebenza iiyure ezinde kunezo uzisebenzayo njengangoku?**	0= Hayi<br>1= Ewe	
E5	**Uqhele ukusebenza unyaka wonke, okanye usebenza ngamaxesha onyaka athile, okanye kube kanye ngelo xesha?**	0= Ukusebenza kube kanye ngelo xesha<br>1= Ukusebenza ngamaxesha onyaka athile<br>2=Ukusebenza unyaka wonke	
E6	**Uyahlawulwa ngomsebenzi owenzileyo, okanye akuhlawulwa kwaphela?**	0= Akuhlawulwa<br>1= Uhlawulwa ngohlobo oluthile kuphela (ufumana izinto /iinkonzo kodwa ingeyomali)<br>2= Uhlawulwa ngemali nangolunye uhlobo<br>3= Uhlawulwa ngemali kuphela	
E7	**Uziva umsebenzi wakho ukhuseleke kangakanani?**	0= Alukho ukhuseleko lomsebenzi<br>1= Lukho ukhuseleko noko<br>2= Ukhuseleko lomsebenzi lusemgangathweni onguwo<br>2= Umsebenzi okhuselekileyo kakhulu	
E8	**Nceda undixelele unobumba ohambelana nentlawulo yakho yaqho ngenyanga kumsebenzi wakho ongundoqo (kubandakanywa ama-ova, izibonelelo, iibhonasi)?**<br><br>**[hand out card, read options before asking question]**	NgeeRandi,<br>A. 0 – 2,000<br>B. 2,001 – 5,000<br>C. 5,001 – 10,000<br>D. 10,001 – 15,000<br>E. 15,001 – 20,000<br>F. Ngaphezu we-20,000	**Code letter here**

77= ndalile 88= andazi (ukuba ayisele idwelisiwe) 97= ayifanelekanga 99= idatha ayikho

Umhla: ___ ___ /___ ___ ___ / ___ ___ ___ ___
DD / MON / Y Y Y Y **Put 5 digit PIN here → __________________**

**Ngoku masithethe ngobomi bakho basekuhlaleni. Nceda undixelele ukuba ngamaxa amangaphi owenze ngawo ezi zinto kwezi nyanga zi-3 zidlulileyo.**<br><br>**[hand out card, read options before asking questions]**

	**Kwezi nyanga zi-3 zidlulileyo, ngamaxa amangaphi...**	**READ CODES OUTLOUD TO PARTICIPANT**<br><br>0=Azange<br>1=Akuxhaphakanga (ndikwenzile oko, kodwa hayi qho)<br>2= Ngamanye amaxesha (ndikwenza oko ngamaxesha ngamaxesha)<br>3= Qho (ndikwenza oko <u>qho ngeveki</u>)<br>4= Qho (ndikwenza <u>oku ngaphezu kwakanye (1) ngeveki</u>)
SL1	**…ukuthenga ukutya okusele kuphekiwe ethakhishophu, okuthengwa kuhanjwe nako okanye erestyu ukulungiselela <u>wena</u>?**	
SL2	**…ukuthenga ukutya okusele kuphekiwe ethakhishophu, okuthengwa kuhanjwe nako okanye erestyu ukulungiselela <u>amalungu osapho</u>?**	
SL3	**…ukuthenga ukutya okusele kuphekiwe ethakhishophu, okuthengwa kuhanjwe nako okanye erestyu ukulungiselela <u>abahlobo bakho</u>?**	
SL4	**…ukuthenga ukutya okusele kuphekiwe ethakhishophu, okuthengwa kuhanjwe nako okanye erestyu ukulungiselela <u>izithandwa zakho</u>?**	
SL5	**…ukuthenga ukutya okusele kuphekiwe ethakhishophu, okuthengwa kuhanjwe nako okanye erestyu ukulungiselela loo <u>mntu umfunayo abe sisithandwa sakho</u>?**	
SL6	**...ukuphisa ngezipho/izinto ngaphandle kokutya /idrinki kumntu othandana naye?**	
SL7	**...ukuphisa ngezipho/izinto ngaphandle kokutya /idrinki kumntu omfunayo ukuba athandana nawe?**	
SL8	**…ukuchitha ixesha ethaveni /eshibhini?**	
SL9	**…ukuchitha ixesha ujikeleza kwiindawo ezineevenkile ezindawonye / ezisaluni zeenwele/ okanye kwiithakhishophu?**	
SL10	**… ukuchitha ixesha udlala iikhadi okanye ungcakaza?**	
SL11	**…ukuchitha ixesha kwiindawo ezingamiselwanga**	

77= ndalile 88= andazi (ukuba ayisele idwelisiwe) 97= ayifanelekanga 99= idatha ayikho

	**zokuhlamba imoto okanye iindawo zepikiniki?**	
SL12	**…ukuzithengela idrinki ethaveni /eshibhini/ kwindawo ekuselwa kuyo utywala?**	
SL13	**…ukuthengela abahlobo bakho idrinki ethaveni /eshibhini/ kwindawo ekuselwa kuyo utywala?**	
SL14	**…ukuthengela umntu omfunayo abe sisithandwa sakho idrinki ethaveni/ eshibhini / kwindawo ekuselwa kuyo utywala?**	

			**CODE**
RIS11	**Kule nyanga idlulileyo, ngokuphakathi nje, uyisele amaxa amangaphi idrinki enotywala?**	0= Awakho<br>1= 1-3 amaxesha ngenyanga<br>2= Qho ngeveki<br>3= Yonke imihla	
RIS12	**Kule nyanga idlulileyo, ngamaxa amangaphi owawunxililе/owasela ngawo isiselo esinxilisayo phambi kokwabelana ngesondo?**	0=Azange<br>1= Ngamanye amaxesha (nganeno kwehafu)<br>2= Kaninzi (ngaphezu kwehafu)<br>3= Qho	
**Abanye abantu bazame iindidi ezahlukeneyo zeziyobisi. Kule nyanga idlulileyo, zeziphi kwezi zilandelayo ukuba zikho, okhe wazisebenzisa?**			
RIS13a	**Khat/ Miraa**	0= Hayi<br>1= Ewe<br>88= andazi	
RIS13b	**Ukuzihlaba iziyobisi ngenaliti**	0= Hayi<br>1= Ewe<br>88= andazi	
RIS13c	**Intsangu**	0= Hayi<br>1= Ewe<br>88= andazi	
RIS13d	**Enye**	0= Hayi<br>1= Ewe<br>88= andazi	
RIS13do	Specify other drug use here →		

Umhla: ___ ___ /___ ___ ___ / ___ ___ ___ ___
DD / MON / Y Y Y Y **Put 5 digit PIN here →** ____________________

[For women only ask:] **Ngoku yeminye imibuzo ngosapho lwakho nembali yenzala. (For males code=97)**

Ngoku yeminye imibuzo ngobom bosapho lwakho…			**CODE**
PG1	**Wakha wakhulelwa?**	0= Hayi<br>1= Ewe	
PG2	**Ukuba ngu-ewe, wawumdala kangakanani xa wawukhulelwa okuqala ngqa?**	Code age as 97 if never pregnant.	
PG3	**Ingaba ukhulelwe ngoku?**	0= Hayi<br>1= Ewe<br>2 = Andiqinisekanga	
PG4<br>DEM210a	**Zingaphi zizonke <u>izisu</u> zethuba eligqithileyo okhe wabanazo?**	WRITE number →<br>USE IAVI rules for NA.	
PG5	**Zingaphi izisu kwezi owawunezicwangciso ngazo?**	WRITE number here→<br>97 if never pregnant.	
PG6<br>DEM210b	**Bangaphi abazalwa bephila owakha waba nabo?**	WRITE number here→<br>USE IAVI rules for NA.	
PG7	**Lalinini ixesha lokugqibela owabeleke ngalo umntwana ophilayo?**	WRITE Mo & Yr here→	_____ /_____<br>**MON / YYYY**
PG8<br>DEM210c	**Bangaphi abantwana bakho abaswelekayo abaphakathi kweminyaka engu-0 no-5 yobudala?**	WRITE number here→<br>USE IAVI rules for NA	
PG9	**<u>Unabantwana</u> abangaphi <u>abangabakho ngqo</u>? Oku kuthetha abantwana abangabakho.**	WRITE number here→	
PG10	**Ingaba wanelisekile ngenani labantwana onalo ngoku?**	1 = Akwanelisekanga kakhulu<br>2 = Akwanelisekanga kancinane<br>3= Akunaluvo / uphakathi nje<br>4 = Wanelisekile<br>5 = Waneliseke kakhulu	
PG11	**Bangaphi abantwana bebonke ongathanda ukuba nabo?**	WRITE number here→	
PG12	**Bangaphi ootata abohlukileyo abangabazali ngqo babantwana bakho?**	WRITE number here→<br>97 if never pregnant.	
PG13	**Kwezi nyanga zi-3 zidlulileyo, ingaba wena okanye iqabane lakho belifuna ukukhulelwa?**<br><br>**[Read options if needed]**	1 = Ndandifuna ukukhulelwa, kodwa iqabane lam lalingafuni<br>2 = Ndandingafuna ukukhulelwa, kodwa iqabane lalifuna<br>3 = Sobabini besifuna<br>4 = Sobabini sasingafuni	
PG14	**Ingaba iqabane lakho okanye wena ufuna ukukhulelwa kunyaka ozayo?**<br><br>**[Read out options]**	1 = Ndiyafuna ukukhulelwa, kodwa iqabane lam alifuni<br>2 = Andifuni ukukhulelwa, kodwa iqabane lam liyafuna<br>3 = Sobabini siyafuna<br>4 = Sobabini asifuni	
PG15	**Buthini ubudala obubububo kumabhinqa ukuze akhulelwe okukuqala ngqa?**	WRITE age here→	

Umhla: ___ ___ /___ ___ ___ / ___ ___ ___ ___
DD / MON / Y Y Y Y **Put 5 digit PIN here → ________________**

PG16	**Buthini ubudala endodeni ukuba mayibe ngutata womntwana?**	WRITE age here→	
PG17	**Wawumdala kangakanani okokuqala ngqa usabelana ngesondo (kwilungu lobufazi okanye kumva)?**	WRITE age here→	

[For men only ask:] Ngoku yeminye imibuzo ngosapho lwakho nembali yenzala. (For females code=97)

			CODE
PG1	**Wakha wakhulelisa umntu obhinqileyo /intombazana?**	0= Hayi<br>1= Ewe<br>2= Andiqinisekanga	
PG2	**Ukuba ngu-ewe, wawumdala kanganani xa wakhulelisa umntu okokuqala ngqa?**	Code 99 if never created pregnancy.	
PG3	**Ingaba iqabane lakho nokuba leliphi na likhulelwe ngoku?**	0= Hayi<br>1= Ewe<br>2 = Andiqinisekanga	
PG4	**Zingaphi izisu ebezizezakho (nokuba isiphumo sibe yintoni na)?**	WRITE number here→<br>97 if never created a pregnancy.	
PG5	**Zingaphi izisu kwezi owawunezicwangciso ngazo?**	WRITE number here→<br>97 if never created a pregnancy.	
PG6	**Bangaphi abazelwe bephilile obungutata kubo ngqo?**	WRITE number here→<br>97 if never created a pregnancy.	
PG7	**Lalinini ithuba lokugqibela owaba ngutata womntwana ophilayo ngalo?**	WRITE Mo&Yr here→	_____ /_____<br>**MON / YYYY**
PG8	**Bangaphi abantwana bakho abaswelekayo abaphakathi kweminyaka engu-0 no-5 yobudala?**	WRITE number here→<br>97 if never fathered a child	
PG9	**<u>Unabantwana</u>** abangaphi **<u>abangabakho ngqo</u>? Oku kuthetha abantwana abangabakho.**	WRITE number here→	
PG10	**Ingaba wanelisekile ngenani labantwana onalo ngoku?**	1 = Akwanelisekanga kakhulu<br>2 = Akwanelisekanga kancinane<br>3= Akunaluvo / uphakathi nje<br>4 = Wanelisekile<br>5 = Waneliseke kakhulu	
PG11	**Leliphi inani olinqwenelayo labantwana?**	WRITE number here→	
PG12	**Bangaphi ootata abohlukileyo abangabazali ngqo babantwana bakho?**	WRITE number here→<br>97 if never fathered a child	
PG13	**Kwezi nyanga zi-3 zidlulileyo, ingaba wena okanye iqabane lakho belifuna ukukhulelwa? [Read options if needed]**	1 = Ndandifuna ukukhulelwa, kodwa iqabane lam lalingafuni<br>2 = Ndandingafuna ukukhulelwa, kodwa iqabane lalifuna<br>3 = Sobabini besifuna<br>4 = Sobabini sasingafuni	
PG14	**Ingaba iqabane lakho okanye wena ufuna ukukhulelwa kunyaka ozayo? [Read out options]**	1 = Ndiyafuna ukukhulelwa, kodwa iqabane lam alifuni<br>2 = Andifuni ukukhulelwa, kodwa iqabane lam liyafuna<br>3 = Sobabini siyafuna	

77= ndalile 88= andazi (ukuba ayisele idwelisiwe) 97= ayifanelekanga 99= idatha ayikho

		4 = Sobabini asifuni.	
PG15	**Buthini ubudala obububo kumabhinqa bokuba akhulelwe okukuqala ngqa?**	WRITE age here→	
PG16	**Buthini ubudala endodeni bokuba mayibe ngutata womntwana?**	WRITE age here→	
PG17	**Wawumdala kangakanani okokuqala ngqa usabelana ngesondo (kwilungu lobufazi okanye kumva)?**	WRITE age here→	

Ngoku ndiza kukubuza ngeenkolelo zakho. Abantu baneenkolelo ezahlukeneyo ezininzi. Okokuqala, ndingathanda ukuva iinkolelo zakho ngeekhondom. Ndiza kukufundela inkcazelo, nceda undixelele ukuba uvumelana okanye akuvumelani kangakanani nale nkcazelo usebenzisa inani kweli khadi. Nceda ukhumbule ukuba akukho zimpendulo zilungileyo nezingalunganga. Nceda undixelele uluvo lwakho.

[Hand out card; explain how to use it]

		**Andivumelani ngamandla**	**Andivumelani**	**Ndiyavumelana**	**Ngamandla Ndiyavumelana**
CU1	**Ndinako ukulicebisa iqabane lam ukuba lisebenzise iikhondom nokuba ngaba alifuni ukwenza oko.**	**1**	**2**	**3**	**4**
CU2	**Ndingakukhumbula ukusebenzisa ikhondom emva kokuba bendisele.**	**1**	**2**	**3**	**4**
CU3	**Ndingema ukuze ndiyifake kum ikhondom okanye kwiqabane lam nokuba ngaba sobabini sivukelwe yinkanuko yokwabelana ngesondo ngokumandla.**	**1**	**2**	**3**	**4**
CU4	**Ndingakwala ukwabelana ngesondo ukuba akukho khondom ifumanekayo.**	**1**	**2**	**3**	**4**
CU5	**Ukuba bendiza kucebisa ngokusebenzisa iikhondom neqabane lam ebendingakhange ndisebenzise iikhondom kunye nalo, ndingaziva ndisoyika ukuba lingandala.**	**1**	**2**	**3**	**4**
CU6	**Ukuba ndicebisa ngokusebenzisa iikhondom kunye neqabane lam elitsha ngoko ke lingacinga ukuba ndinesifo sokwabelana ngesondo.**	**1**	**2**	**3**	**4**
CU7	**Ukuba ndicebisa ngokusebenzisa iikhondom kunye neqabane lam elitsha ngoko ke lingacinga bendicinga ukuba linesifo sokwabelana ngesondo.**	**1**	**2**	**3**	**4**

Ngoku yimibuzo emalunga neenkolelo zakho ezimalunga nokukhulelwa. Nceda undixelele ukuba uvumelana okanye akuvumelani kangakanani nezi nkcazelo zingezantsi. [Repeat instructions and reminders]

	**[Provide response card]**	**Andivumelani ngamandla**	**Andivumelani**	**Ndiyavumelana**	**Ngamandla Ndiyavumelana**
PB1	**Intombazana mayikhulelwe ukubonisa ukuba inako ukufumana abantwana.**	1	2	3	4
PB2	**Indoda iya kufuna ukuyitshata kuphela intombazana ukuba iyenze yakhulelwa yiyo ngaphambili.**	1	2	3	4

77= ndalile 88= andazi (ukuba ayisele idwelisiwe) 97= ayifanelekanga 99= idatha ayikho

Umhla: ___ ___ /___ ___ ___ / ___ ___ ___ ___
DD / MON / Y Y Y Y **Put 5 digit PIN here → ____________________**

	[Provide response card]	Andivumelani ngamandla	Andivumelani	Ndiyavumelana	Ngamandla Ndiyavumelana
PB3	**Intombazana asingomntu obhinqileyo ngaphandle kokuba ikhe yanomntwana.**	1	2	3	4
PB4	**Inkwenkwe ayiyondoda ide ibe ikhe yaba nomntwana nomntu obhinqileyo.**	1	2	3	4
PB5	**Lihlazo kusapho ukuba intombazana iyakhulelwa phambi kokuba itshate.**	1	2	3	4
PB6	**Indoda iya kufuna ukutshata intombazana ukuba ikhe yazala umntwana/abantwana.**	1	2	3	4
PB7	**Abazali bafumana ilobola encinci ukuba intombi ibikhe yakhulelwa yaba nomntwana.**	1	2	3	4
PB8	**Amabhinqa ayakhulelwa ukwenzela ukuba afumane igranti karhulumente yabantwana.**	1	2	3	4
PB9	**Umfazi ukholelwa ukuba makakhulelwe ukubonisa ukuba ungumfazi ngokwenene.**	1	2	3	4
PB10	**Abahlobo banefuthe omnye komnye malunga nokuba bakhulelwe.**	1	2	3	4
PB11	**Ukukhulelwa luphawu lokuba ngumfazi.**	1	2	3	4
PB12	**Amadoda akholelwa ekubeni umfazi makakhulelwe ukubonisa ukuba ungumfazi ngokwenene.**	1	2	3	4

77= ndalile 88= andazi (ukuba ayisele idwelisiwe) 97= ayifanelekanga 99= idatha ayikho

Umhla: ___ ___ /___ ___ ___ / ___ ___ ___ ___
DD / MON / Y Y Y Y **Put 5 digit PIN here → ________________**

Ngoku yimibuzo emalunga neenkolelo zakho ngobudlelwane phakathi kwamadoda nabafazi.

	**[Provide response card]**	**Ngamandla Andivumelani**	**Ndiyavumelana**	**Andivumelani**	**Ndiyavumelana ngamandla**
R1	Amadoda athanda izithandwa ezininzi kuba kusendalweni kubo ukwenza ngolo hlobo	1	2	3	4
R2	Amadoda anezithandwa zokuba afumane amandla okwanelisa amaqabane awo angundoqo	1	2	3	4
R3	Abafazi kule mihla bathi kufuneka babe nangaphezu kweqabane elinye lezesondo	1	2	3	4
R4	Amadoda aziva eneentloni ngabafazi bawo yaye afuna izithandwa zabo ezilulutsha abanozisa kubahlobo bazo	1	2	3	4
R5	Ukuba amadoda awanazo izithandwa abahlobo bazo bayabahleka	1	2	3	4
R6	Abafazi abazimeleyo ngokwezemali abafuni kuzibophelela kubudlelwane obunye	1	2	3	4
R7	Iintsapho zabantu abatsha abasebenzayo azifuni ukuba batshate kuba boyika ukuphulukana nengeniso yabo	1	2	3	4
R8	Amadoda asoloko enyanzela abafazi ngeendlela ezithile ukuba babelane nabo ngesondo nokuba abafuni	1	2	3	4
**Umyeni unazo izizathu zokumbetha umfazi wakhe ukuba…**					
PWR1	…uyahamba engamxelelanga.	1	2	3	4
PWR2	…akabahoyi abantwana.	1	2	3	4
PWR3	…uxoxa kunye naye.	1	2	3	4
PWR4	… uyala ukwabelana naye ngesondo.	1	2	3	4
PWR5	…utshisa ukutya.	1	2	3	4

Okukugqibela, eminye imibuzo ngeendlela zokuthintela iHIV.

		**CODE**<br>0= Hayi<br>1= Ewe<br>2= Andiqinisekanga
K1	Ingaba kungokunje kukho isigonyo esinqanda abantu ekubeni bosuleleke yiHIV?	
K2	Ingaba kungokunje kukho isigonyo esithothisa isifo emva kokuba abantu bosuleleke yiHIV?	
K3	Ingaba kungokunje kukho ijel abafazi abayifakayo kumalungu abo obufazi nenqanda ukufumana iHIV ngethuba lokwabelana ngesondo?	
K4	Ukuba indoda iye yeluswa kubudoda bayo ngugqirha kunokwenzeka ukuba yosuleleke yiHIV?	

Esi sisiphelo sodliwano-ndlebe. Enkosi ngokuvuma ukuthatha ixesha lakho uphendule le mibuzo yethu.

TIMEEND	**Ixesha lokuPhela koDliwano-ndlebe**	HH:MM using 24 hour clock	___ ___ : ___ ___

[Refer as needed. Correct knowledge as needed using text below]:

- Ndifuna ukuqinisekisa ukuba uyazi ukuba akukho sigonyo sokunqanda okanye sokuthothisa iHIV.

Umhla: ___ ___ /___ ___ ___ / ___ ___ ___ ___
DD / MON / Y Y Y Y **Put 5 digit PIN here → ___________________**

- Ingaba kungokunje kukho ijel abafazi abayifakayo kumalungu abo obufazi nenqanda ukufumana iHIV ngethuba lokwabelana ngesondo.
- Izigonyo neejel ziyavavanywa eMzantsi Afrika ukufumanisa ukuba ziyasebenzana. Kodwa ngoku asinaso isigonyo okanye ijel esebenzayo.
- Amadoda alusiweyo kubudoda bawo ngugqirha asengozini engabhekanga phi yokosuleleka yiHIV. Ndingakunika iinkcukacha zoqhagamshelwano zikagqirha uwandolusayo.

Umhla: ___ ___ /___ ___ ___ / ___ ___ ___ ___
DD / MON / Y Y Y Y **Put 5 digit PIN here →** ___________________

INFORMATION TO BE COMPLETED BY INTERVIEWER AFTER INTERVIEW:

				CODE
I1	**Interviewer code for interviewer**			
I2	**In what language was the interview administered?**	0=English<br>1=Tswana<br>2=Afrikaans<br>3=Mix of English & Tswana<br>4=Xhosa<br>5=Zulu<br>6=Other		
I2o		If other specify→		

INFORMATION TO BE COMPLETED BY STUDY COORDINATOR OR DESIGNEE:

		**CODE**
**Is the participant eligible for IAVI Protocol B?**	0= Ineligible<br>1= Eligible<br>97= Not applicable	
