# Supplementary material for: HIV incidence and predictors of inconsistent condom use among adult men enrolled into an HIV vaccine preparedness study, Rustenburg, South Africa
Source: PLoS One. 2019 Apr 3;14(4):e0214786. doi: 10.1371/journal.pone.0214786 (PMC6447216; doi:10.1371/journal.pone.0214786)
Supplement: S6 File — (PDF) [file pone.0214786.s008.pdf]

Date: ___ ___ /___ ___ ___ / ___ ___ ___ ___
DD / MON / Y Y Y Y **Put 5 digit PIN here → _______________**

Start time: ________:_______
hh mm

AURUM CLINICAL ASQ

**Kwezi nyanga zi-3 zidlulileyo…**			**CODE**
STI1	**…ingaba ugqirha okanye unesi ukufumanise unaso nasiphi na isifo kwezo zosulela ngokwabelana ngesondo ekuthwa zii-STI?**	0= Hayi<br>1= Ewe	
STI2	**…ukhe wanyangelwa nasiphi na isifo esiyi-STI?**	0= Hayi<br>1= Ewe	
STI3<br>RIS14	**…ingaba ukhe wanencindi enukayo okanye engaqhelekanga ephuma kwilungu lobufazi okanye lobudoda bakho?**	0= Hayi<br>1= Ewe	
STI4	**…ungaba kukhe kwabuhlungu xa uchamayo?**	0= Hayi<br>1= Ewe	
STI5	**…ingaba kukhe kwabuhlungu xa usabelana ngesondo?**	0= Hayi<br>1= Ewe	
STI6<br>RIS15	**…ingaba ukhe wanazo naziphi na izilonda kwilungu lobufazi okanye lobudoda?**	0= Hayi<br>1= Ewe	
STI7	**IF FEMALE…have you had any lower abdominal pain?<br>Code=97 if male**	0= Hayi<br>1= Ewe	
STI8	**IF FEMALE: …ukhe watshotshozelelwa kwilungu lobufazi lakho okanye kwatshisa kulo?<br>Code=97 if male**	0= Hayi<br>1= Ewe	

ASK OF MEN ONLY (if female code = 97)

**Ngoku yimibuzo emalunga nokoluswa kwakho kubudoda bakho—apho kususwa khona ijwabi lakho.**			**CODE**
CIR1	**Ingaba wakha woluswa kubudoda bakho ngugqirha okanye ngunesi?**	0= Hayi<br>1= Ewe	
CIR2	**[If yes, circumcised by doctor/nurse]<br>Wawumdala kangakanani xa wawusoluswa ngugqirha okanye ngunesi?**	Code age in years	
CIR3	**[If no, not medically circumcised]<br>Ingaba uyafuna ukuthunyelwa kugqirha onako ukukolusa?**	0= Hayi<br>1= Ewe	
CIR4	**Wakha woluswa ebudodeni bakho ngokwesiko lakwaNtu?**	0=Hayi<br>1=Ewe	
CIR5	**[If circumcised in cultural practice]<br>Wawumdala kangakanani xa wawusoluswa ngokwesiko lakwaNtu?**	Code age in years	

77=ndalile 88= andazi (ukuba ayisele idwelisiwe) 97= ayifanelekanga 99= idatha ayikho

Date: ___ ___ /___ ___ ___ / ___ ___ ___ ___

DD / MON / Y Y Y Y **Put 5 digit PIN here → _______________**

Ngoku ndiza kukubuza ngezinto wena neqabane lakho eninokuba nizisebenzisile kwezi nyanga zi-3 zidlulileyo. Ukuba ubuneqabane elinye kwezi nyanga zi-3 zidlulileyo, nceda ucinge ngaloo mntu xa uphendula le mibuzo. Ukuba ubunamaqabane angaphezu kwesinye kwezi nyanga zi-3 zokugqibela, nceda ucinge ngeqabane owawusabelana nalo ngesondo ubukhulu becala.

	**Ndiza kukufundela kolu luhlu. Nceda undixelele ukuba iqabane lakho likhe lasebenzisa nayiphi indlela kwezi zilandelayo kwiinyanga ezi-3 ezidlulileyo...**	**CODES**<br>0=Hayi<br>1=Ewe<br>88=Andazi<br>97= ayifanelekenga, akukho lwabelwano ngesondo
C1	**i-injekshini yokucwangcisa yeehomoni (idepo)**	
C2	**iipilisi zokucwangcisa eziziihomoni (ipilisi)**	
C3	**ndasikhupha isisu**	
	**Nceda undixelele ukuba iqabane lakho likhe lasebenzisa nayiphi indlela kwezi zilandelayo amaxesha amangaphi kwiinyanga ezi-3 ezidlulileyo...**	**CODES**<br>0=Azange<br>1=Ngamanye amaxesha<br>2= kwihafu yeli xesha<br>3= Ubuninzi beli xesha<br>4=Phantse lonke elo xesha<br>5= Ngokuqinisekileyo qho ngexesha elinye<br>97= ayifanelekenga, akukho lwabelwano ngesondo
C4	**iikhondom zamadoda**	
C5	**iikhondom zabafazi**	
C6	**Wabukhupha ubudoda phambi kokuchama, okanye wayikhuphela ngaphandle kwelungu lobufazi imbewu yobudoda**	

	**Ingaba wena okanye iqabane lakho lenza oku …**	**CODES**<br>0=Hayi<br>1=Ewe
C7	**ukubotshwa kwemibhobho (umfazi ovalwa inzala okanye ukubotshwa kwemibhobho)**	
C8	**ukukhutshwa kwesizalo (uqhaqho lokukhutshwa kwesibeleko sobhinqileyo)**	
C9	**uqhaqho lokuvala inzala kumadoda (amadoda enziwe amadlolo)**	

	**Ngoku ndiza kukubuza ngokuba ngamaxa amangaphi owabelene ngesondo ngawo kwiveki edlulileyo oko kukuthi kwezi ntsuku zisi-7 zidlulileyo.**		**CODE**
LW1	**Mangaphi amaxesha okhe wabelana ngesondo ngawo kwezi ntsuku zisi-7 zidlulileyo, kubandakanywa nonamhlanje?**	Code number of sex acts →	
LW2	**Ingaba eli linani lezenzo zokwabelana ngesondo kwilungu lobufazi kwiveki enye yesiqhelo (umlinganiselo wakho wesiqhelo)?**	0= Hayi<br>1= Ewe	
LW3	**Mangaphi amaxesha okhe wabelana ngesondo ngomva ngawo kule veki idlulileyo?**	Code number of sex acts →	

77=ndalile 88= andazi (ukuba ayisele idwelisiwe) 97= ayifanelekanga 99= idatha ayikho

LW4	**Ingaba eli linani lezenzo zokwabelana ngesondo ngomva kwiveki enye yesiqhelo (umlinganiselo wakho wesiqhelo)?**	0= Hayi<br>1= Ewe	

Date: ___ ___ /___ ___ ___ / ___ ___ ___ ___
DD / MON / Y Y Y Y **Put 5 digit PIN here → _______________**

			CODE
	**ASK THESE OF PEOPLE WHO SAID THEY HAVE PULLED OUT THE PENIS IN LAST 3 MOS**		
CIY1	**Yayimbono kabani ukubukhupha ubudoda?**	0= Imbono yam<br>1= Imbono yeqabane<br>2= Zombini<br>3= Akunjalo-kwasuka kwenzeka<br>4= Enye	
CIY2	**If code other above Write reason for pulling out here →**		
CIY3	**Ingaba wena neqabane lakho nathetha ngokumalunga nokubukhupha ubudoda bakho phambi okanye ngethuba lokwabelana ngesondo?**	0= Hayi<br>1= Ewe	
CIY4	**IF YES – Tell me how this topic came up, and what was discussed →**		
CIY5	**Kwakutheni ukuze wena okanye iqabane lakho libukhuphe ubudoda?**<br><br>**[DO NOT READ ANSWERS OUT ALOUD – LET PARTICPANT ANSWER FIRST. USE OTHER IF NECESSARY]**	0= Ukuthintela iHIV<br>1= Ukuthintela ii-STIs<br>2= Ukuthintela ukukhulelwa<br>3= Ukuthintela zombini ukukhulelwa nezifo<br>4= Andazi ukuba kwakutheni- kwasuka kwenzeka<br>5= Izizathu zezenkcubeko – cacisa apha ngezantsi<br>6= Enye	
CIY6	**If coded as 'cultural' or other above write reason for pulling out here →**		
CIY7	**Lithuba elingakanani uneliqabane (othe wabukhupha ubudoda bakho ukunye nalo?)**	Ikhowudi ngeenyanga kwixesha lilonke lidibene (ungalibali ixesha ningekho kunye).<br>Code 0 if one time partner →	
	**Ixesha lokugqibela ukhuphe ubudoda bakho, nceda undixelele nantoni na enye owawuyisebenzisa kwangelo xesha linye…**		**CODES**<br>0=Hayi<br>1=Ewe
CIY8	**i-injekshini yokucwangcisa yeehomini (idepo)**		
CIY9	**iipilisi zokucwangcisa eziziihomoni (ipilisi)**		
CIY10	**iikhondom zamadoda**		
CIY11	**iikhondom zabafazi**		
CIY12	**…kwesintu**		
CIY13	**If traditional specify →**		
CIY14	**…enye? Cacisa→**		

CIY15	**Ingaba esi yayisisizathu oqhele ukusisebenzisa xa ukhupha ubudoda bakho?**		

**ASK THESE OF PEOPLE WHO SAID THEY HAVE NOT PULLED OUT THE PENIS IN LAST 3 MOS**			**CODE**
CIN1	**Ngoba kutheni? Write reason for not using pulling out penis here →**		
CIN2	**Ingaba wakha wasebenzisa ukubukhupha ubudoda bakho?**	0= Hayi<br>1= Ewe	
CIN3	**[IF YES TO EVER USE] Ndixelele ukuba yavela njani le ncoko, yaye naxoxa ngantoni**		
CIN4	**[IF YES TO EVER USE]<br>Kwakutheni ukuze wena okanye iqabane lakho libukhuphe ubudoda?<br><br>[DO NOT READ ANSWERS OUT LOUD – LET PARTICPANT ANSWER FIRST]**	0= Ukuthintela iHIV<br>1= Ukuthintela ii-STIs<br>2= Ukuthintela ukukhulelwa<br>3= Ukuthintela zombini ukukhulelwa nezifo<br>4= Andazi ukuba kwakutheni-kwasuka kwenzeka<br>5= Enye	
CIN5	**[If code other above write reason for pulling out here] →**		
CIN6	**Lithuba elingakanani uneliqabane (othe wabukhupha ubudoda bakho kulo ukunye nalo)?**	Ikhowudi ngeenyanga kwixesha lilonke lidibene (ungalibali ixesha ningekho kunye).<br>Code 0 if one time partner. →	
CIN7	**Wawumdala kangakanani kwixesha lokugqibela lokubukhupha ubudoda bakho?**	Code age here →	

Date: ___ ___ /___ ___ ___ / ___ ___ ___ ___
DD / MON / Y Y Y Y **Put 5 digit PIN here → _______________**

ASK THIS PAGE TO ALL PARTICIPANTS – NO MATTER WHAT THEIR PRACTICE IS – THERE ARE NO RIGHT OR WRONG ANSWERS HERE – THESE MEASURE PARTICIPANT BELIEFS –NOT PRACTICES

Ngoku ndingathanda ukuva ngeenkolelo zakho ngokubukhupha ubudoda phambi kokuba uchame, okanye ukhuphele imbewu yobudoda ngaphandle. [Repeat instructions and reminders]

	**[Provide response card]**	**Andivumelani ngamandla**	**Andivumelani**	**Ndiyavumelana**	**Ngamandla Ndiyavumelana**
WDMP	Bekukho amaxesha ebendifuna ukubukhupha ubudoda phambi kokuchama neqabane (namaqabane) lam lesiqhelo /neqabane (namaqabane) eliphambili	**1**	**2**	**3**	**4**
WDCP	Bekukho amaxesha ebendifuna ukubukhupha ubudoda phambi kokuchama neqabane (namaqabane) lam lamaxesha athile	**1**	**2**	**3**	**4**
WD1	Ndinako ukulicebisa iqabane lam ukuba lilikhuphe ilungu lobudoda (iimbewu yobudoda ibengaphandle) nokuba alifuni ukwenza oko.	**1**	**2**	**3**	**4**
WD2	Ndingakukhumbula ukulikhupha ilungu lobudoda phambi nasemva kokuba bendisela utywala.	**1**	**2**	**3**	**4**
WD3	Ndingakuqinisekisa ukuba ndiyalikhupha ilungu lobudoda (imbewu ibe yobudoda ibe ngaphandle) nokuba sobabini sivukelwe sanenkanuko yokwabelana ngesondo.	**1**	**2**	**3**	**4**
WD4	Ndingala ukwabelana ngesondo ukuba akukho thuba lokulikhupha ilungu lobudoda.	**1**	**2**	**3**	**4**
WD5	Ukuba bendinokucebisa ngokuba likhutshwe ilungu lobudoda (imbewu yobudoda ibe ngaphandle) neqabane endingazange ndikwenze oko nalo ngaphambili, ndingaziva ndisoyika ukuba iqabane lam lingandala.	**1**	**2**	**3**	**4**
WD6	Ukuba ndicebisa ngokulikhupha ilungu lobudoda iqabane lam lingacinga ukuba ndinezifo zokwabelana ngesondo.	**1**	**2**	**3**	**4**
WD7	Ukuba ndicebisa ngokulikhupha ilungu lobudoda iqabane elitsha lingacinga ukuba ndicinge ukuba linesifo sokwabelana ngesondo.	**1**	**2**	**3**	**4**
WD8	Ukuba ndicebisa ngokulikhupha ilungu lobudoda iqabane lam lingacinga ukuba andifuni kukhulelwa.	**1**	**2**	**3**	**4**
WD9	Ukuba bendiza kulikhupha ilungu lobudoda phambi kokuchama, iqabane lam lingacinga ukuba bendizama ukuthintela <u>ukufumana</u> iHIV.	**1**	**2**	**3**	**4**
WD10	Ukuba bendingalikhupha ilungu lobudoda phambi kokuchama, iqabane lam lingacinga ukuba bendizama <u>ukususela</u> abanye nge-HIV.	**1**	**2**	**3**	**4**
WD11	Ukuba bendingalikhupha ilungu lobudoda, iqabane lam lingacinga ukuba andikwazi ukuwugqiba umsebenzi.	**1**	**2**	**3**	**4**
WD12	Ukuba bendingalikhupha ilungu lobudoda, iqabane lam	**1**	**2**	**3**	**4**

77=ndalile 88= andazi (ukuba ayisele idwelisiwe) 97= ayifanelekanga 99= idatha ayikho

	lingandihleka				
WD13	Ukuba bendingalikhupha ilungu lobudoda, iqabane lam lingacinga ukuba bendiyindoda enamandla	**1**	**2**	**3**	**4**
WD14	Ukuba bendingalikhupha ilungu lobudoda, iqabane lam lingacinga ukuba ndiyakwazi ukuzilawula ngokuyimpumelelo.	**1**	**2**	**3**	**4**
WD15	Ukuba bendingalikhupha ilungu lobudoda, iqabane lam lingacinga ukuba andimava kwezokwabelana ngesondo.	**1**	**2**	**3**	**4**

Date: ___ ___ /___ ___ ___ / ___ ___ ___ ___
DD / MON / Y Y Y Y **Put 5 digit PIN here → _______________**

			CODE
RIS16	**Kwezi <u>nyanga zi-3 zidlulileyo</u>, mangaphi kumaqabane owabelane nawo ngesondo lawo abe ngamadoda?**	Enter no. of <u>male</u> partners →	
RISMCMFRQ	**Kwezi nyanga zi-3 zidlulileyo, ngamaxa amangaphi osebenzise ngawo iikhondom namaqabane angamadoda akho?**	0=Azange<br>1= Ngamanye amaxesha (nganeno kwehafu yeli xesha)<br>2= Kaninzi (ngaphezu kwehafu yeli xesha)<br>3= Qho<br>4= Akubuzwanga (Akukho maqabane amangamadoda)	
RIS16a	**Kula ___ maqabane mangaphi abengamaqabane amatsha, oko kukuthi umntu ongazange wabelane ngesondo naye ngaphambili?**	Enter no. of <u>new male</u> partners →	
RIS16b	**Wazisebenzisa amaxa amangaphi iikhondom xa wawusabelana ngesondo nala maqabane ma- ____ matsha angamadoda?**	0=Azange<br>1= Ngamanye amaxesha (nganeno kwehafu yeli xesha)<br>2= Kaninzi (ngaphezu kwehafu yeli xesha)<br>3= Qho<br>4= Akubuzwanga (Akukho maqabane amangamadoda)	
RIS17	**<u>Kwezi nyanga zi-3 zidlulileyo,</u> mangaphi kumaqabane owabelane nawo ngesondo lawo abe ngabafazi?**	Enter no. of <u>female</u> partners →	
RISMCFFRQ	**<u>Kwezi nyanga zi-3 zidlulileyo</u>, ngamaxa amangaphi osebenzise ngawo iikhondom namaqabane angabafazi akho?**	0=Azange<br>1= Ngamanye amaxesha (nganeno kwehafu yeli xesha)<br>2= Kaninzi (ngaphezu kwehafu yeli xesha)<br>3= Qho<br>4= Akubuzwanga (akukho maqabane angabafazi)	
RIS17a	**Kula ___ maqabane mangaphi abengamaqabane amatsha, oko kukuthi umntu ongazange wabelane ngesondo naye ngaphambili?**	Enter no. of <u>new female</u> partners →	
RIS17b	**Wazisebenzisa amaxa amangaphi iikhondom xa wawusabelana ngesondo nala maqabane ma- ____ matsha angamadoda?**	0=Azange<br>1= Ngamanye amaxesha (nganeno kwehafu yeli xesha)<br>2= Kaninzi (ngaphezu kwehafu yeli xesha)<br>3= Qho<br>4= Akubuzwanga (akukho maqabane angabafazi)	
RIS28	**Mangaphi kula maqabane ma ____angamadodoa nangabafazi abene-HIV?**	0= Hayi<br>1= Ewe → if yes, go to next Q<br>88= Andazi	
PAR2	**Ingaba ukho kula oliqabane elosuleleke yi-HIV- othatha ii-ARVs nokuba zeziphi na (amayeza eHIV) kwezi nyanga zi-3 zidlulileyo?**	0= Awakho<br>1= Abanye<br>2= Onke<br>88= Andazi	
RIS28b	**Wazisebenzisa amaxa amangaphi**	0=Azange	

77=ndalile 88= andazi (ukuba ayisele idwelisiwe) 97= ayifanelekanga 99= idatha ayikho

	**iikhondom xa wawusabelana ngesondo nala maqabane ane-HIV ?**	1= Ngamanye amaxesha (nganeno kwehafu yeli xesha)<br>2= Kaninzi (ngaphezu kwehafu yeli xesha)<br>3= Qho<br>4= Akubuzwanga (akukho maqabane osuleleke yi-HIV)	
RIS29	**Kula maqabanema- ___ owabelana nawo ngesondo mangaphi *owafumana* imali kuwo, izipho okanye uncedo ukwenzela ukuba wabelana nawo ngesondo?** (ngezipho okanye uncedo ndithetha ukuhlawula iintlawulo, izithuthi, ukutya,imizuzu yokufona, iimpahla, imali okanye nayiphi na enye intlawulo okanye engenziwanga ngexesha elifana nelo lokwabelana ngesondo.)	Code number →	
RIS210	**Kula maqabane ma- ___ owabelana nawo ngesondo, mangaphi *owawanika* imali, izipho okanye uncedo ukwenzela ukuba wabelana nawo ngesondo?** (ngezipho okanye uncedo ndithetha ukuhlawula iintlawulo, izithuthi, ukutya,imizuzu yokufona, iimpahla, imali okanye nayiphi na enye intlawulo okanye engenziwanga ngexesha elifana nelo lokwabelana ngesondo.)	Code number →	
RIS211	**Kwezi nyanga zi-3 zidlulileyo, ingaba wakha wanyanzeliswa ukuba wabelane ngesondo ungafuni ukwenza oko?**	0= Hayi<br>1= Ewe → refer to supervisor for f/up	
RIS212	**Kwezi nyanga zi-3 zidlulileyo, ukhe wahamba okanye wahlala kude nekhaya kaninzi?** (ngaphezu kobusuku obu-3 evekini ngeveki ngokuphakathi)	0= Hayi<br>1= Ewe	
RIS213	**Kwezi nyanga zi-3 zidlulileyo ingaba wakha wazibandakanya kulwabelwano ngesondo niliqela, oko kukuthi, ukwabelana ngesondo kunye nangaphezu komntu omnye ngaxeshanye?**	0= Hayi<br>1= Ewe, abafazi kuphela<br>2= ewe, amadoda kuphela<br>3= Ewe, amadoda nabafazi<br>4= Akubuzwanga	
RIS214	**Kwezi nyanga zi-3 zidlulileyo ingaba ukhe wabelana ngesondo ngomva ungaphantsi apho iqabane lakho lifaka ubudoda balo kuwe ngemva?**	0= Hayi<br>1= Ewe<br>2= Akubuzwanga	
RIS214a	**Wazisebenzisa amaxa amangaphi iikhondom xa usabelana ngesondo ungaphantsi ngomva apho iqabane lakho lifaka ilungu lobudoda kumva wakho?**	0=Azange<br>1= Ngamanye amaxesha (nganeno kwehafu yeli xesha)<br>2= Kaninzi (ngaphezu kwehafu yeli xesha)<br>3= Qho<br>4= Akubuzwanga (akukho kwabelana ngesondo ngomva)	
RIS215	**MEN ONLY: Kwezi nyanga zi-3 zidlulileyo ingaba ukhe wabelana ngesondo ngomva ungaphezulu apho wena ufaka ubudoda bakho**	0= Hayi<br>1= Ewe<br>2= Akubuzwanga	

	**kumva weqabane lakho?**		
RIS215	**MEN ONLY: Ngamaxa amangaphi owasebenzisa ngawo ikhondom xa wawusabelana ngesondo ungaphezulu ngomva oko kukuthi, xa ufaka ilungu lobudoda kumva waqabane lakho?**	0=Azange<br>1= Ngamanye amaxesha (nganeno kwehafu yeli xesha)<br>2= Kaninzi (ngaphezu kwehafu yeli xesha)<br>3= Qho<br>4= Akubuzwanga (akukho kwabelana ngesondo ngomva)	

77=ndalile 88= andazi (ukuba ayisele idwelisiwe) 97= ayifanelekanga 99= idatha ayikho

Date: ___ ___ /___ ___ ___ / ___ ___ ___ ___
DD / MON / Y Y Y Y **Put 5 digit PIN here →** _______________

COLLECT FROM ALL: VITAL SIGNS (Nurse, enrolled nurse, doctor or clinician to take vitals)

			CODE
V0	**Who took these vitals?**	0=Unesi obhaliswe ngokusemthethweni<br>1= Unesi oqeqeshiweyo<br>2 = Ugqirha<br>3= Enye	
V1BP<br>PEX11	**Blood pressure**	record mm/Hg→	_ _ _ / _ _ _
V2P<br>PEX12	**Pulse**	record beats per minute→	
VRR<br>PEX13	**Respiration Rate**	record breaths per minute →	_ _
VBT<br>PEX14	**Body Temperature**	record to 1 decimal place in degrees Celcius (XX.X) →	_ _ . _
V5 W<br>PEX15	**Weight**	record in kg→	_ _ _
V6H<br>PEX16	**Height**	record in cm→	_ _ _

TO BE COMPLETED BY THE NURSE/DOCTOR, BASED ON PROFESSIONAL JUDGEMENT

		EWE	HAYI
ELI1 9	**Ubukho bayo nayiphi na imeko enokuphazamisana nokufikelela kwiinjongo zophando?**		

____
Staff code

End time: _______:______
hh mm

Umhla: ___ ___ /___ ___ ___ / ___ ___ ___ ___
DD / MON / Y Y Y Y
